# Intracellular delivery of Parkin-RING0-based fragments corrects Parkin-induced mitochondrial dysfunction through interaction with SLP-2

**DOI:** 10.1186/s12967-024-04850-3

**Published:** 2024-01-16

**Authors:** Alessandra Zanon, Marianna Guida, Alexandros A. Lavdas, Corrado Corti, Maria Paulina Castelo Rueda, Alessandro Negro, Peter P. Pramstaller, Francisco S. Domingues, Andrew A. Hicks, Irene Pichler

**Affiliations:** 1 Institute for Biomedicine, Eurac Research, Affiliated Institute of the University of Lübeck, Bolzano, Italy; 2 Department of Biomedical Sciences, University of Padova, Padua, Italy; 3 Department of Neurology, University Medical Center Schleswig-Holstein, Campus Lübeck, Lübeck, Germany

**Keywords:** Parkinson′s disease, Parkin, SLP-2, Mitochondria, Parkin mini-peptide

## Abstract

**Background:**

Loss-of-function mutations in the *PRKN* gene, encoding Parkin, are the most common cause of autosomal recessive Parkinson’s disease (PD). We have previously identified mitoch

ondrial Stomatin-like protein 2 (SLP-2), which functions in the assembly of respiratory chain proteins, as a Parkin-binding protein. Selective knockdown of either Parkin or SLP-2 led to reduced mitochondrial and neuronal function in neuronal cells and *Drosophila*, where a double knockdown led to a further worsening of Parkin-deficiency phenotypes. Here, we investigated the minimal Parkin region involved in the Parkin-SLP-2 interaction and explored the ability of Parkin-fragments and peptides from this minimal region to restore mitochondrial function.

**Methods:**

In fibroblasts, human induced pluripotent stem cell (hiPSC)-derived neurons, and neuroblastoma cells the interaction between Parkin and SLP-2 was investigated, and the Parkin domain responsible for the binding to SLP-2 was mapped. High resolution respirometry, immunofluorescence analysis and live imaging were used to analyze mitochondrial function.

**Results:**

Using a proximity ligation assay, we quantitatively assessed the Parkin-SLP-2 interaction in skin fibroblasts and hiPSC-derived neurons. When PD-associated *PRKN* mutations were present, we detected a significantly reduced interaction between the two proteins. We found a preferential binding of SLP-2 to the N-terminal part of Parkin, with a highest affinity for the RING0 domain. Computational modeling based on the crystal structure of Parkin protein predicted several potential binding sites for SLP-2 within the Parkin RING0 domain. Amongst these, three binding sites were observed to overlap with natural PD-causing missense mutations, which we demonstrated interfere substantially with the binding of Parkin to SLP-2. Finally, delivery of the isolated Parkin RING0 domain and a Parkin mini-peptide, conjugated to cell-permeant and mitochondrial transporters, rescued compromised mitochondrial function in Parkin-deficient neuroblastoma cells and hiPSC-derived neurons with endogenous, disease causing *PRKN* mutations.

**Conclusions:**

These findings place further emphasis on the importance of the protein–protein interaction between Parkin and SLP-2 for the maintenance of optimal mitochondrial function. The possibility of restoring an abolished binding to SLP-2 by delivering the Parkin RING0 domain or the Parkin mini-peptide involved in this specific protein–protein interaction into cells might represent a novel organelle-specific therapeutic approach for correcting mitochondrial dysfunction in Parkin-linked PD.

**Supplementary Information:**

The online version contains supplementary material available at 10.1186/s12967-024-04850-3.

## Background

Parkinson’s disease (PD) is an age-related, progressive neurodegenerative disorder, characterized by a selective loss of dopaminergic (DA) neurons in the midbrain *substantia nigra pars compacta*. The subsequent dopamine deficiency in the basal ganglia triggers cellular and synaptic dysfunction, leading to the classical parkinsonian motor symptoms, which include tremor, rigidity, bradykinesia (slowing of movement), and postural instability accompanied by non-motor symptoms [1]. The underlying causes of the DA neuron degeneration are not completely resolved, but the identification of rare variants in a number of genes linked to familial forms of PD have allowed a better understanding of the pathogenic mechanisms leading to both familial and sporadic PD [2–4]. Many familial PD genes are associated either directly or indirectly with mitochondrial homeostasis, thus highlighting a crucial role of mitochondrial impairment in DA neuron degeneration [5, 6], particularly in early stage PD highlighting the need of disease-modifying strategies tailored to early disease [7, 8]. Loss-of-function mutations in the *PRKN* gene encoding Parkin are the most common known cause of early-onset autosomal recessive PD, accounting for up to 42.2% of cases with an age of onset ≤ 20 years [9]. Parkin dysfunction also represents a risk factor for sporadic PD [10, 11]. *PRKN* variants include a wide spectrum of rearrangements and copy number variations, such as deletions and multiplications of exons, small indels, as well as single nucleotide polymorphisms causing nonsense, missense or splice site mutations [2, 12]. Such pathogenic variants have been shown to stop the translation of a functional protein or to affect protein folding and/or protein–protein interactions and recruitment to mitochondria [13, 14]. Parkin is a RING-in-between-RING E3 ubiquitin ligase comprising an N-terminal ubiquitin-like (Ubl) domain, followed by zinc-coordinating domains RING0 (also known as Unique Parkin Domain, UPD), RING1, an In-Between-RING (IBR) domain, a linker domain named repressor element of Parkin (REP), and the RING2 domain [15, 16]. It is localized in the cytoplasm and translocates to the mitochondria upon specific stimulation [17, 18]. A portion of endogenous Parkin has been suggested to be localized within mitochondria [19]. Following Parkin recruitment to mitochondria and activation by phosphorylation, outer mitochondrial membrane proteins are ubiquitinated, and components of the autophagy machinery are recruited, leading to the selective autophagic removal of the damaged organelle, a process known as mitophagy [18, 20–22]. This process was shown to be impaired also in *PRKN*-PD patient-derived neurons [23, 24]. Additionally, Parkin is involved in the activation of mitochondrial biogenesis through ubiquitination and subsequent degradation of PARIS (Parkin interacting substrate), a transcriptional repressor of the master regulator of mitochondrial biogenesis PGC1-α (peroxisome proliferator-activated receptor gamma coactivator 1α) [25, 26]. More recently, it was shown that mitochondrial dysfunction in human DA neurons lacking Parkin was primarily due to defects in mitochondrial biogenesis largely driven by the upregulation of PARIS [24]. Furthermore, *PRKN*-patient neurons revealed deficits in mitochondrial biogenesis linked to metabolic modulation of the Sirtuin1/PGC1-α pathway and resulting in mtDNA dyshomeostasis and neuroinflammation [27]. Suppression or knockout of *PRKN* in *Drosophila*, zebrafish, mice and human patient cells leads to decreased ATP production, complex I activity, an altered mitochondrial morphology, locomotor impairments, and neuronal loss [28–32]. Parkin is also implicated in mitochondrial dynamics, increased lifespan, reduced proteotoxicity, and sequestration of reactive dopamine metabolites [33, 34]. We have previously reported that Parkin interacts with mitochondrial Stomatin-like protein 2 (SLP-2) [35, 36], and that induced overexpression of SLP-2 can correct mitochondrial functional alterations caused by Parkin-deficiency, in neuronal cells from human origin and in *Drosophila,* thus suggesting a protective role in neurons [36]. SLP-2 acts as a scaffold protein in the inner mitochondrial membrane, facilitating both the assembly of respiratory chain complexes and their function [37–39]. Moreover, inclusion of SLP-2 in a mitochondrial protease complex (SPY) has an important role in determining the form and function of mitochondria as well as the regulation of cell signaling and survival [40].

Protein–protein interactions (PPIs) are mediated by peptide sequences and can be mimicked by peptidomimetics. Notably, it has been estimated that 15–40% of all PPIs are enabled through short, linear stretches of peptide sequence [41], and peptides designed based on the sequences involved in the interaction can mask or substitute a critical domain of the binding site. In addition, peptides can be chemically modified to stabilize the bioactive conformation mimicking the 3D protein structure. Further, peptides and peptidomimetics can modulate intracellular targets by crossing the cell membrane independently or via conjugation to cell-penetrating vehicle peptides [42–44].

In this study, we further investigated the Parkin-SLP-2 interaction and found that the RING0 domain of Parkin is critical for the binding to SLP-2. Expression of this isolated domain and a smaller Parkin peptide identified within this domain by bioinformatic approaches was able to restore mitochondrial malfunction in Parkin-deficient cells.

## Materials and methods

### Cell lines

Skin fibroblasts were obtained from the University of Lübeck (Prof. Christine Klein), the Neuro-Biobank of the University of Tübingen, and the Telethon Network of Genetic Biobanks [45] under MTA. Human iPSCs from two control individuals (iPS-802 and iPS-SFC084-03-02) and two PD patients carrying a homozygous mutation in the *PRKN* gene (iPS-2011 and iPS-B125) were included in the study. iPS-802 and iPS-2011 were generated in the laboratory of the Institute for Biomedicine by using a protocol we have published previously [46, 47]. iPS-B125 was generated for a previous study [36] and shared by the University of Lübeck (Prof. Christine Klein); iPSC line SFC084-03-02 was established through the StemBANCC consortium (https://cells.ebisc.org/STBCi033-B/). The study was approved by the Ethics Committee of the South Tyrolean Health Care System (approval number 102/2014 dated 26.11.2014 with extension dated 19.02.2020).

### Cell culture

Human skin fibroblasts, human neuroblastoma (SH-SY5Y, ATCC CRL-2266) and HeLa (ATCC CCL-2) cells were cultured in Dulbecco’s modified Eagle’s Medium (DMEM, Sigma) supplemented with 10% fetal bovine serum and 1% penicillin–streptomycin (Thermo Fisher Scientific). SH-SY5Y cells with a stable knockdown of Parkin were produced as described previously [36]. Cells were maintained at 37 °C in a saturated humidity atmosphere containing 5% CO_2_. Human induced pluripotent stem cells (hiPSCs) were differentiated into DA neurons as described previously [36, 48–50]. In brief, human iPSC colonies were disaggregated into single cells using accutase and replated onto matrigel (BD)-coated dishes in mTeSR^™^ 1 complete medium, supplemented with 10 μM ROCK inhibitor Y-27632 (Miltenyi Biotech) at a density of 57,000–62,000 cells/cm^2^. Differentiation was started once hiPSCs reached a confluence of 90% by adding knockout serum replacement (KSR) medium supplemented with SMAD pathway inhibitors SB431542 (SB, Miltenyi Biotech) and LDN-193189 (LDN, StemMACS). On days 1 to 5, KSR medium was added to the cells in the presence of SB, LDN, recombinant Human Sonic Hedgehog (SHH, R&D System), recombinant Human FGF-8a (FGF8, R&D System), and Purmorphamine (Pu, StemMACS). The Wnt pathway activator molecule CHIR99021 (CH, StemMACS) was included from days 3–12. During days 6–10 of differentiation, increasing amounts of Neurobasal medium plus B27 supplement (NB-B27 medium, Thermo Fisher Scientific) was added to the KSR medium (25, 50, and 75%), and upon day 7, SHH, FGF8, and Pu were withdrawn. On day 11, maturation of DA neurons was initiated by adding recombinant Human BDNF (Peprotech), ascorbic acid (Sigma Aldrich), recombinant Human TGF-ß3 (Peprotech), cyclic-AMP (EnzoLifescience) and DAPT (Tocris).

To dissipate the mitochondrial membrane potential, cells were exposed to the protonophore carbonyl cyanide m-chlorophenylhydrazone (CCCP) (10 µM, Sigma-Aldrich).

### Co-immunoprecipitation (co-IP)

Human neuroblastoma SH-SY5Y and HeLa cells were harvested and resuspended in 1 ml lysis buffer (150 mM NaCl, 50 mM Tris–HCl pH 7.6, 1% NP-40, 0.1% SDS, supplemented with protease and phosphatase inhibitors, Roche Diagnostics). Lysates were incubated on ice for 30 min and cleared by centrifugation at 14,200 × g for 20 min. Then, samples were equalized for the protein concentration using the DC Protein assay (Biorad) and precleared by incubation with protein A agarose beads (Roche Diagnostics) for 30 min at 4 °C. The beads were removed by centrifugation, and the samples were incubated overnight at 4 °C on a rotator with rabbit anti-HA (Santa Cruz, sc-805) or control IgG (purified rabbit IgG, Millipore). 50 µl of protein A agarose beads were added to the samples, followed by incubation for 2 h on a rotator at 4 °C. Next, the beads were pelleted by centrifugation, and the supernatant was discarded. The beads were washed three times with washing buffer (150 mM NaCl, 50 mM Tris–HCl, 1% Igepal, pH 7.6), and then proteins were released from the beads by heating at 95 °C for 5 min in 4X Sample buffer containing DTT (Thermo Fisher Scientific), followed by sodium dodecyl sulphate polyacrylamide gel electrophoresis (SDS-PAGE), blotting and incubation with mouse anti-SLP-2 antibody (Abcam ab89025, 1:1000).

### Western blot (WB) analysis

SDS-PAGE was performed using NuPAGE 4–12% Bis–Tris gels (Thermo Fisher Scientific). After electrophoresis, proteins were transferred onto a nitrocellulose membrane (Biorad) and probed with antibodies raised against mouse anti-Parkin (Cell Signaling, mAb #4211, 1:1000), mouse anti-SLP-2 (Abcam, ab89025, 1:1000), rabbit anti-HA (Santa Cruz, sc-805, 1:500), mouse anti-β-actin (Sigma Aldrich, A5316, 1:5000), mouse anti-tubulin (Abcam, ab44928, 1:5000), mouse anti-GAPDH (Millipore, MAB374, 1:500). Immunoreaction was visualized using the SuperSignal West Dura Chemiluminescence Westernblot Substrate (Thermo Fisher Scientific).

### Generation of human recombinant Parkin and SLP-2 proteins

Recombinant human Parkin proteins (aa 1–456 and aa 138–465) and human SLP-2 protein (amino acids, aa 41–356) were produced in fusion with SUMO protein in One Shot™ BL21(DE3)pLysS Chemically Competent E. coli (Thermo Fisher Scientific) and purified on Immobilized-metal affinity chromatography (IMAC) columns under nondenaturing conditions. SUMO protein was cleaved with ULP-1 protease.

### Far-Western blot analysis

2 µg of recombinant Parkin proteins (aa 1–465 and aa 138–465) were separated in SDS-PAGE, transferred onto immobilon membrane (Millipore) and incubated for 6 h with 80 µg of recombinant SLP-2. After exhaustive washing, the membrane was probed with an anti-SLP-2 antibody (Abcam ab89025, 1:1000). Recombinant SLP-2 (2 µg) was loaded, transferred, and incubated with 60 µg of recombinant Parkin before probing with an anti-Parkin antibody (Cell Signaling, mAb #4211, 1:1000).

### Proximity ligation assay

To visualize the interaction between Parkin and SLP-2 in fixed cells, the Proximity Ligation Assay (PLA) technique (Duolink kit, Merck, DUO92101) was used [51]. Cells were fixed with 4% paraformaldehyde and permeabilized using a 0.5% Triton-X-100 solution in PBS and subsequently a 0.05% Tween 20 solution in TBS. Cells were blocked for 30 min at 37 °C using the Duolink blocking solution and incubated with the primary antibodies (mouse anti-Parkin, Cell Signaling, mAb #4211, 1:1000; rabbit anti-Parkin, Novus Biologicals, NBP1-67660, 1:500; mouse anti-SLP-2 antibody (Abcam ab89025, 1:1000); rabbit anti-SLP-2, Adipogen, AG-25B-0019, 1:1000; rabbit anti-HA, Santa Cruz, sc-805, 1:500) overnight at 4 °C. Then, cells were washed twice for 5 min in buffer A and incubated with the oligonucleotide-conjugated secondary antibodies for 1 h at 37 °C, followed by the addition of the ligation solution, so that the two oligonucleotides hybridized and joined to a closed circle when they were in close proximity (< 40 nm). Next, the amplification solution was added, initiating a rolling-circle-amplification reaction to generate a concatemeric DNA strand onto which the fluorescent detection probes hybridized, resulting in the tagging of areas, where the two proteins are in close proximity. Finally, samples were covered with glass coverslips using DAPI-containing mounting medium and imaged using a Leica SP8-X confocal microscope (Leica Microsystems).

### Generation of DNA constructs and transient transfections

N- and C-terminal Parkin fragments as well as Ubl, RING0, RING1, and RING2 domains were amplified from the mammalian expression vector encoding human Parkin, pRK5-HA-Parkin, obtained from Addgene. Respective primer sequences are listed in Additional file 1: Table S1. The PCR products were cloned into the Not I and SalI sites of the pRK5-HA vector. Site-directed mutagenesis in the Parkin RING0 domain to insert P153A, K161A and K211A pathogenic PD-causing mutations was performed using the QuikChange site-directed mutagenesis kit (Agilent, 200523). All constructs were validated by Sanger sequencing. GFP-tagged mitochondrial leading sequence (mito-GFP) and GFP-tagged LC3b (lyso-GFP) were purchased from Thermo Fisher Scientific. SH-SY5Y and HeLa cells were transfected using Fugene HD according to the manufacturer’s instructions (Promega).

### Immunofluorescence

For immunocytochemical analysis, cells were fixed in a 4% paraformaldehyde solution in PBS for 10 min at room temperature (RT), permeabilized in a 0.5% Triton-X-100 solution in PBS for 5 min and blocked for 1 h with 3% BSA in PBS. Immunostaining was performed by addition of the primary antibodies (mouse anti-HA, Sigma Aldrich, H3663, 1:1000; rabbit anti-GRP75, Abcam, ab53098; 1:5000; goat anti-c-myc, Novus Biologicals, NB600335, 1:500) in the blocking solution overnight at 4 °C and the appropriate fluorescently labelled secondary antibodies for 1 h at RT. Nuclei were stained by addition of DAPI. Images were acquired using a Leica SP8-X confocal microscope (Leica Microsystems) and analyzed using Imaris software (Oxford Instruments).

### Synthesis of Parkin mini-peptides

A Parkin mini-peptide was designed spanning 11 amino acids over the binding residue in RING0 overlapping with the PD-causing mutation p.K211N mutation (FFKCGAHPTSD) and subsequently synthesized at the Cribi Peptide Facility (University of Padova). It was synthesized in three different versions, conjugated either to i) a cell-permeant C-terminal TAT sequence (RKKRRQRRR) [52] (peptide#1), ii) a C-terminal Fx* r Fx* K Fx* r Fx* K sequence, which was described as cell-permeable peptide able to enter mitochondria [53] (peptide#3), or iii) a D*R*–Dmt*–Orn*–F–NH2 sequence that acts as mitochondrial transporter [54] (peptide#4). The peptides were purified by high resolution preparative HPLC on 6 µm C18 column, and the correct molecular weight was verified by MALDI TOF/TOF spectrometry. The Parkin mini-peptide was applied to cells for 16 h, the concentration depends on the peptide conjugate used (peptide#1: 20 µM, pepide#3: 2.5 µM, peptide#4: 0.5 µM).

### Tripartite split GFP assay

The tripartite split GFP assay was performed in *E. coli* according to published protocols [55]. The assay is based on tripartite association between two twenty amino-acids long GFP tags (GFP10 and GFP11), fused to both Parkin and SLP-2 proteins, and the complementary GFP1-9 detector. When proteins interact, GFP10 and GFP11 self-associate with GFP1-9 to reconstitute a functional GFP. For this assay, Parkin-10, Parkin-11, SLP-2-10, and SLP-2-11 were cloned in pRSET B vector (Thermo Fisher Scientific). In addition, pCDFDuet-1 plasmid (Novagen) was used to co-express GFP1-9 with Parkin-10, Parkin-11, SLP-2-10, SLP-2-11.

### High-resolution respirometry

High-resolution respirometry was performed using the Oxygraph 2 K (Oroboros Instruments, Innsbruck, Austria). Respiration rates were calculated as the time derivative of oxygen concentration measured in the closed respirometer, expressed per million viable cells and corrected by non-mitochondrial oxygen consumption. The amplified signal of the calibrated oxygen concentration and oxygen flux was recorded using the DatLab software for data acquisition and analysis (Oroboros Instruments). Oxygen consumption was measured in cells permeabilized with digitonin (8 μg/ml) in MiRo5 medium (10 mM KH_2_PO_4_, 60 mM lactobionic acid, 20 mM HEPES, 3 mM MgCl_2_, 0.5 mM EGTA, 20 mM taurine, 110 mM D-Sucrose and 1 mg/ml BSA fatty acid free) at final cell densities of 1 × 10^6^ cells/ml. The following parameters were assessed: (1) physiological respiratory activity in intact cells (routine respiration); (2) maximal capacity of the respiratory chain, ETS (non-physiological maximal uncoupled respiration that is not limited by the enzyme activity of the ATP synthase) after induction by stepwise titration of carbonyl cyanide p-(trifluoromethoxy) phenylhydrazone, FCCP (0,5 µM); (3) complex I—dependent respiration after addition of ADP, malate and glutamate, and (4) residual oxygen consumption, ROX (respiration attributable to other cellular oxygen-consuming processes besides the respiratory chain) after addition of 2.5 µM antimycin A. ROX was subtracted from the other parameters. All reagents were purchased from Sigma-Aldrich.

### Oxygen consumption rate

Measurement of oxygen consumption rate (OCR) in SH-SY5Y cells was performed by using the fluorescence-based Extracellular O2 Consumption Assay Kit (Abcam, ab197243) as described previously [50]. In brief, 2.5–2.8 × 10^4^ cells/well were seeded on 96-well plates. Cells recovered for 24 h prior to the treatment with the peptides for 16 h. Immediately after performing the OCR assay, the CyQuant^™^ proliferation assay (Thermo Fisher Scientific, C35012) was employed to determine the number of live cells in each well, according to the manufacturer’s instructions. OCR calculated for each well was normalized to the cell number determined by CyQuant fluorescence dye. Fluorescence intensities detecting OCR are expressed as relative fluorescence units (RFU) versus time (min).

### Live-cell imaging

Mitochondrial superoxide detection was performed by incubating cells in HBSS containing 2.5 µM MitoSox Red mitochondrial superoxide indicator (Thermo Fisher Scientific) for 15 min at 37 °C and 5% CO_2_. Mitochondrial membrane potential was measured using 80 nM TMRM mitochondrial membrane potential indicator (Thermo Fisher Scientific) for 30 min at 37 °C and 5% CO_2_. After incubation, cells were washed three times with HBSS. iPSC-derived neurons were imaged at day 40 of differentiation. Images were acquired in live conditions using a Leica SP8-X confocal microscope (Leica Microsystems) and analyzed using Imaris software (Oxford Instruments).

### Computational analysis of Parkin-binding sites

The individual RING0 domain structure was used for investigating potential binding sites, assuming that the domain structure is mostly preserved and only the domain orientation is affected by the conformational changes. A sequence-based method (ISIS) was applied to the full-length Parkin sequence (Uniprot O60260), and three structure-based methods (PROMATE, SPPIDER, PreDUs) were applied to the structural model PDB ID 4I1H chain A [56] that includes human Parkin domains RING0 to RING2, and to PDB 1IYF chain A [57] that includes the Ubl domain.

### Statistical analysis

Statistical analyses were performed using GraphPad Prism 9. One-way ANOVA was used in experiments comparing at least three groups, followed by appropriate post-hoc tests for pairwise comparisons. For analyzing differences between two experimental groups, the unpaired Mann Whitney U test was utilized. Threshold for significance was set at *p* < 0.05. All experiments were performed in at least three independent biological replicates.

## Results

### Parkin and SLP-2 interact directly and *PRKN* mutations interfere with the interaction

**Fig. 1:**
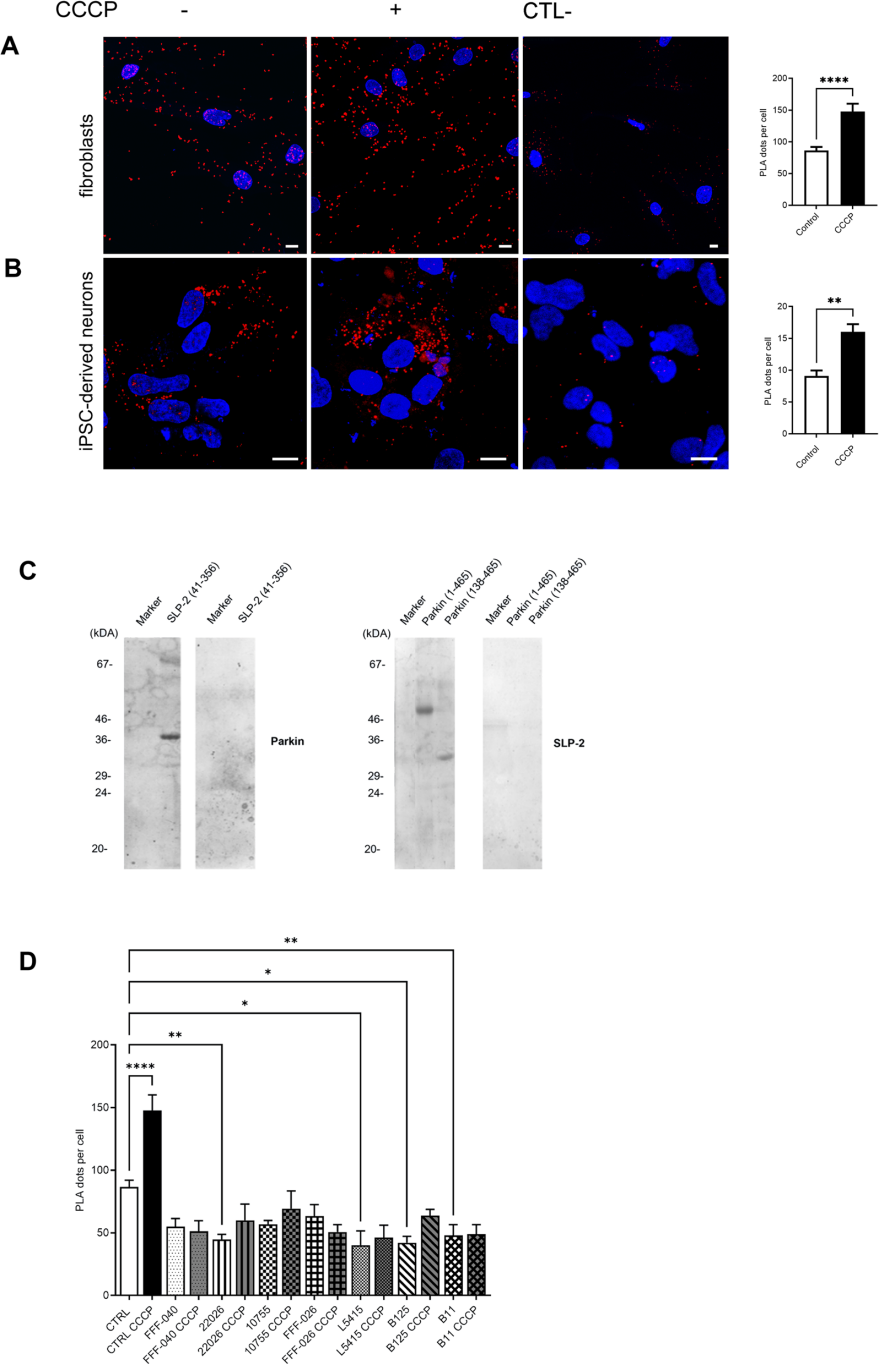
Parkin and SLP-2 interact in human control fibroblasts and hiPSC-derived neurons. **A** Representative PLA staining for fibroblasts of a control individual under normal culture conditions and after CCCP treatment (3 h, 10 µM). The PLA signal is visualized in red, DAPI-stained nuclei are shown in blue. Scale bar: 10 µm. Quantification of PLA dots per cell shows a significantly higher PLA signal after CCCP exposure, indicating an increased interaction between the two proteins. Statistical differences were calculated by unpaired Mann Whitney U test ****p ≤ 0.0001. **B** hiPSC-derived neurons of a control individual were processed using PLA under normal culture conditions and after CCCP treatment (3 h, 10 µM). Scale bar: 10 µm. Quantification of PLA dots per cell shows a significantly higher PLA signal after CCCP exposure. Statistical differences were calculated by unpaired Mann Whitney U test. **p ≤ 0.005. The specificity of the PLA interaction results was confirmed by performing the experiments with only one of the two primary antibodies. **C** Far-Western blot analysis using recombinant SLP-2 (aa 41–356) and Parkin proteins (aa 1–465 and aa 138–465) shows the direct binding of the two proteins. **D** Quantification of PLA dots per cell in control and *PRKN* mutant fibroblast lines under normal culture conditions and after CCCP treatment (3 h, 10 µM) using an antibody directed against the Parkin N-terminus and an anti-SLP-2 antibody. Only in the control lines, CCCP treatment resulted in a significantly increased interaction. Statistical differences were calculated by one-way ANOVA followed by Tukey’s post hoc test to correct for multiple comparisons. * ≤ 0.05, **p ≤ 0.01, ****p ≤ 0.0001

We have previously detected an interaction of Parkin with SLP-2 by tandem affinity purification [35], which we have confirmed in SH-SY5Y cells by immunoprecipitation assays [36]. Here, we further investigated this interaction with endogenous levels of the proteins, using PLA in human control skin fibroblasts and hiPSC-derived DA neurons, with antibodies recognizing Parkin and SLP-2. These experiments demonstrated a close proximity of the proteins in response to mitochondrial uncoupling with CCCP, which triggers Parkin translocation to the mitochondria, whereas under basal conditions, this interaction was significantly reduced but not completely absent, suggesting some Parkin localization at the mitochondria under baseline physiological conditions (Fig. 1A, B). To investigate if the binding between the two proteins is direct or requires additional proteins in a multiprotein complex, we performed a Far-Western blot analysis using recombinant SLP-2 and Parkin proteins. SLP-2 protein was immobilized on a nitrocellulose membrane and probed with full-length human Parkin protein. The protein complex was detected by an anti-Parkin antibody and was absent in the negative control with no Parkin protein. Immobilized recombinant Parkin protein (both full-length and a fragment comprising amino acids, aa 138–465) also directly bound SLP-2 (Fig. 1C). This result was further supported by the association of the recombinant Parkin and SLP-2 proteins observed by using the tripartite split GFP method in *E. coli* (Additional file 1: Fig. S1).

The effect of different *PRKN* mutations on the Parkin-SLP-2 binding was investigated in human fibroblast lines carrying naturally occurring PD-causing mutations located in the N- and C-terminus of the Parkin protein (Additional file 1: Table S2). By using PLA with an antibody directed against the Parkin N-terminus, for all seven *PRKN* mutant fibroblast lines a significantly reduced interaction between the mutant Parkin forms and SLP-2 was detected as compared to the control line. The treatment with CCCP led to a significantly increased interaction only for wildtype Parkin in the control line, but for none of the tested mutant forms, suggesting their inability to translocate to mitochondria (Fig. 1D).

### SLP-2 preferentially binds the Parkin RING0 domain

**Fig. 2:**
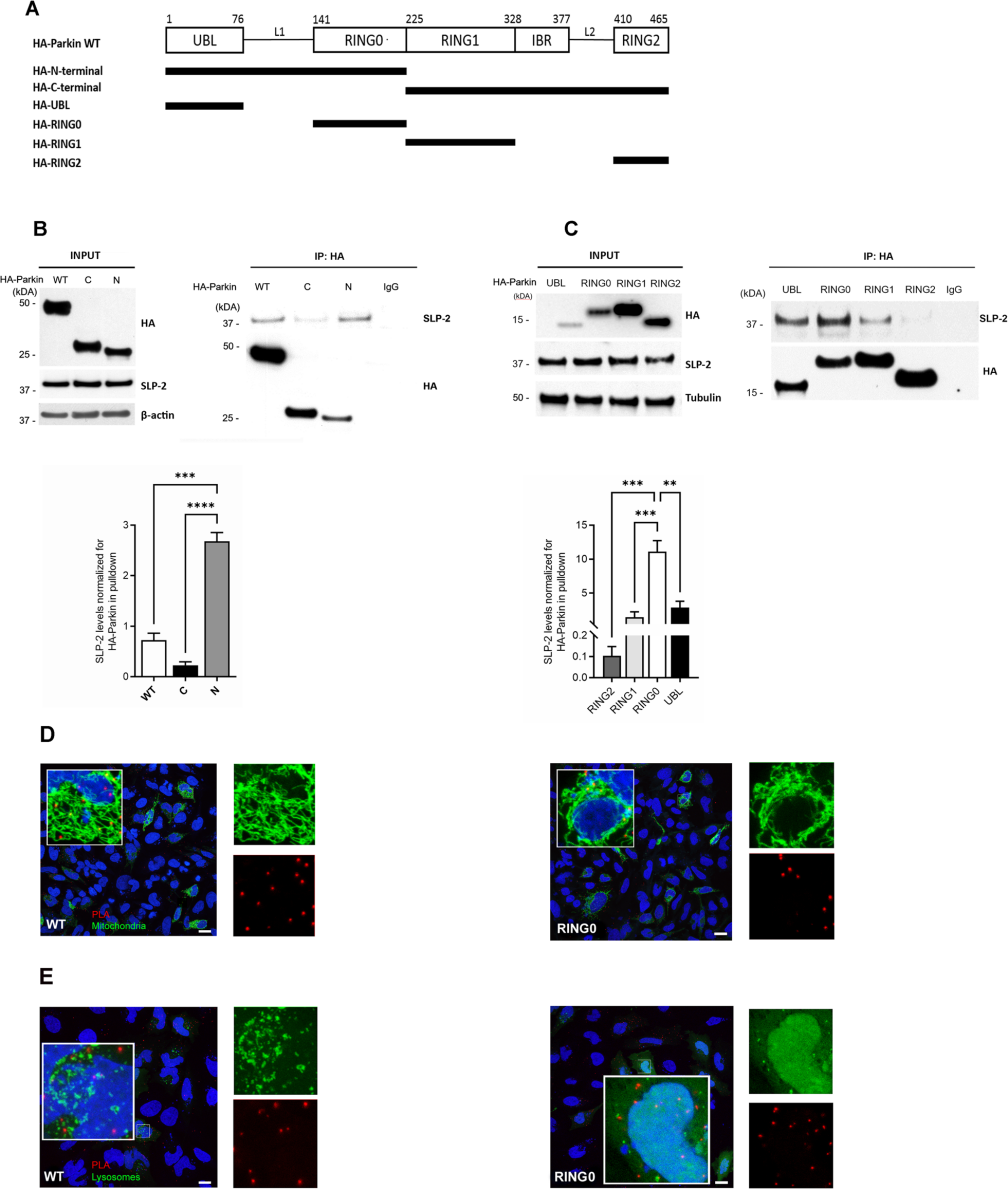
SLP-2 preferentially binds to the Parkin RING0 domain. **A** Overview of the Parkin fragments cloned in PARK5-HA mammalian expression vector. **B** Whole cell lysates of SH-SY5Y cells transfected with wild type (WT), C-terminal (C), or N-terminal (N) Parkin-HA and myc-tagged SLP-2 were subjected to co-IP using an anti-HA antibody, followed by Western blot analysis (WB) of input and IP fractions with the indicated antibodies. Statistical differences were calculated by one-way ANOVA followed by Tukey’s post hoc test to correct for multiple comparisons ***p ≤ 0.001, ****p ≤ 0.0001. **C** Whole cell lysates of SH-SY5Y cells transfected with UBL-, RING0-, RING1-, or RING2-HA and SLP-2-myc were subjected to co-IP using an anti-HA antibody, followed by WB analysis of input and IP fractions with the indicated antibodies. Statistical differences were calculated by one-way ANOVA followed by Tukey’s post hoc test to correct for multiple comparisons. **p ≤ 0.01, ***p ≤ 0.001. **D** Co-stainings of a PLA experiment for Parkin RING0-HA and SLP-2 with a mitochondrial and **E** a lysosomal marker show that the interaction signal co-localizes with the mitochondria but not with the lysosomes. Scale bar: 20 µm (**D**); scale bar: 10 µm (**E**)

To map the Parkin domain responsible for the binding to SLP-2, SH-SY5Y cells were transfected with N-terminally HA-tagged full-length Parkin (HA-Parkin) or N- and C-terminally HA-tagged deletion mutants (HA-N-terminal: aa 1 to 224; HA-C-terminal: aa 225 to 456) (Fig. 2A). Co-immunoprecipitation of the full-length Parkin and the N- or C- terminal parts of Parkin with endogenous SLP-2 revealed that the N-terminal Parkin fragment is sufficient for the SLP-2 interaction (Fig. 2B). Furthermore, HA-tagged constructs of isolated Parkin domains (UBL, RING0, RING1 and RING2) were generated, and co-immunoprecipitation experiments showed a preferential binding of SLP-2 to the RING0 domain (aa 145 to 225), contained within the N-terminal Parkin fragment. The UBL, RING1 and RING2 domains displayed some, but lower, binding capability (Fig. 2C). These results were further corroborated by the Far-Western blot analysis performed for the recombinant Parkin protein lacking only the UBL domain (aa 138–465), which was still able to bind SLP-2 (Fig. 1C).

When transiently expressing the isolated RING0 Parkin domain and the RING0 domain linked to a mitochondrial targeting sequence in HeLa cells, which have little or no endogenous Parkin expression [58, 59], immunofluorescence staining showed a partial localization of the RING0 domain in the vicinity of mitochondria, which was visible without mitochondrial membrane depolarization by CCCP exposure and increased when using the mitochondrial targeting sequence (Additional file 1: Fig. S2). To verify that the interaction of the transfected Parkin-RING0 fragment with endogenous SLP-2 occurs at the mitochondria, PLA for HA-Parkin RING0 and endogenous SLP-2 was combined with the transfection of a GFP-tagged mitochondrial leading sequence (mito-GFP) (Fig. 2D). A co-staining of the PLA experiment with a lysosomal marker (GFP-tagged LC3b, lyso-GFP) suggests that the Parkin RING0-fragment is not immediately degraded by the lysosomes (Fig. 2E).

### Fine-mapping of the amino acid residues in Parkin RING0 involved in SLP-2 binding

**Fig. 3:**
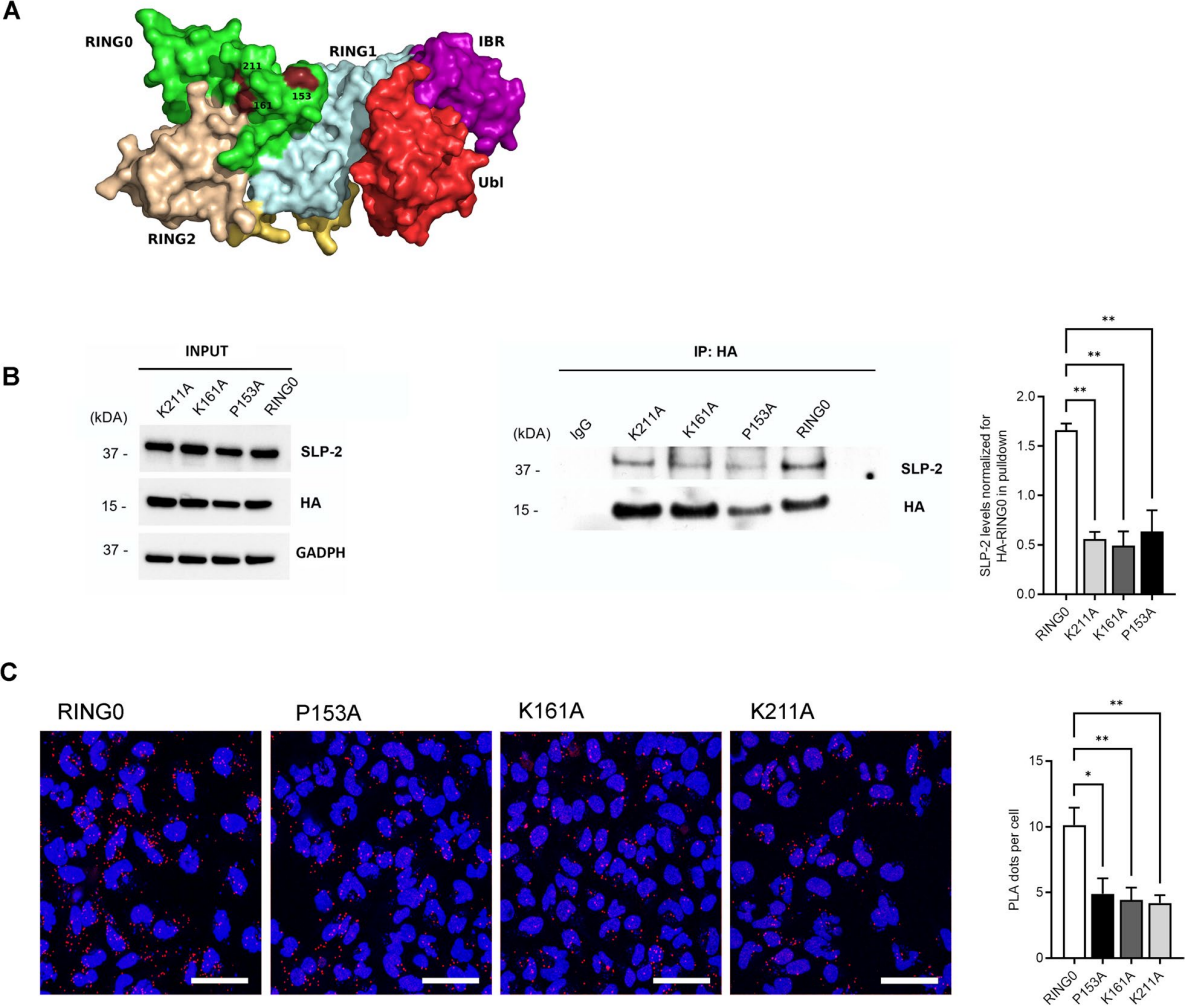
Pathogenic mutations in Parkin RING0 interfere with binding to SLP-2. **A** Representation of the three-dimensional structure of human Parkin showing the Parkin domains. **B** Whole cell lysates of SH-SY5Y cells transfected with RING0 Parkin-HA and three mutant RING0 fragments (P153A, K161A and K211A) were subjected to co-IP using an anti-HA antibody, followed by WB analysis of input and IP fractions with the indicated antibodies. Statistical differences were calculated by one-way ANOVA followed by Tukey's post hoc test to correct for multiple comparisons **p ≤ 0.01. **C** HeLa cells were processed using PLA after transfection with RING0-HA and the mutant RING0 domains using anti-HA and anti-SLP-2 antibodies. For all three mutant RING0 fragments with one of the three missense mutations, a decreased interaction was detected. The PLA signal is visualized in red, DAPI-stained nuclei are shown in blue. Scale bar: 50 µm. Statistical differences were calculated by one-way ANOVA followed by Tukey’s post hoc test to correct for multiple comparisons *p ≤ 0.05, **p ≤ 0.01

Next, we aimed to identify the minimal protein interaction sites within Parkin RING0 responsible for the interaction with SLP-2. To identify candidate binding sites, several bioinformatic prediction methods (ISIS [60], PROMATE [61], SPPIDER [62] and PreDUs [63]) were used. The amino acid candidate residues were prioritized based on the number of consensus predictions, an overlap with known PD-linked mutations, and the exposure of the residues in the 3D protein structure. Nine identified candidate binding sites in Parkin RING0 overlap with PD-causing mutations (Additional file 1: Table S3), and the three most prioritized candidate binding residues overlap with the missense mutations p.P153R, p.K161N, and p.K211N [64–66] (Fig. 3A). These three PD-linked mutations were inserted separately in the RING0 fragment by site-directed mutagenesis. Whole cell lysates of SH-SY5Y cells transfected with HA-Parkin RING0 and the three mutant HA-RING0 fragments were subjected to co-immunoprecipitation, followed by the detection of SLP-2. For all three mutations in RING0, a reduced binding to SLP-2 was detected (Fig. 3B). To further evaluate these findings, we performed PLA experiments in HeLa cells transfected with full-length RING0 or the three mutant RING0 fragments and again observed a significant reduction of the binding capability of the mutant RING0 fragments as compared to wildtype RING0 (Fig. 3C). Moreover, mutant RING0 fragments colocalized with the mitochondria to the same extent as wildtype RING0, and no colocalization with the lysosomes was detected (Additional file 1: Fig. S3), indicating that these fragments are partially present at the mitochondria after transfection. These data suggest that the protein binding sites at amino acid residues 153, 161, and 211 in Parkin RING0, together, or individually, are involved in the binding of Parkin to SLP-2.

### Expression of Parkin RING0 domain rescued impaired mitochondrial respiration in Parkin-deficient cells

**Fig. 4:**
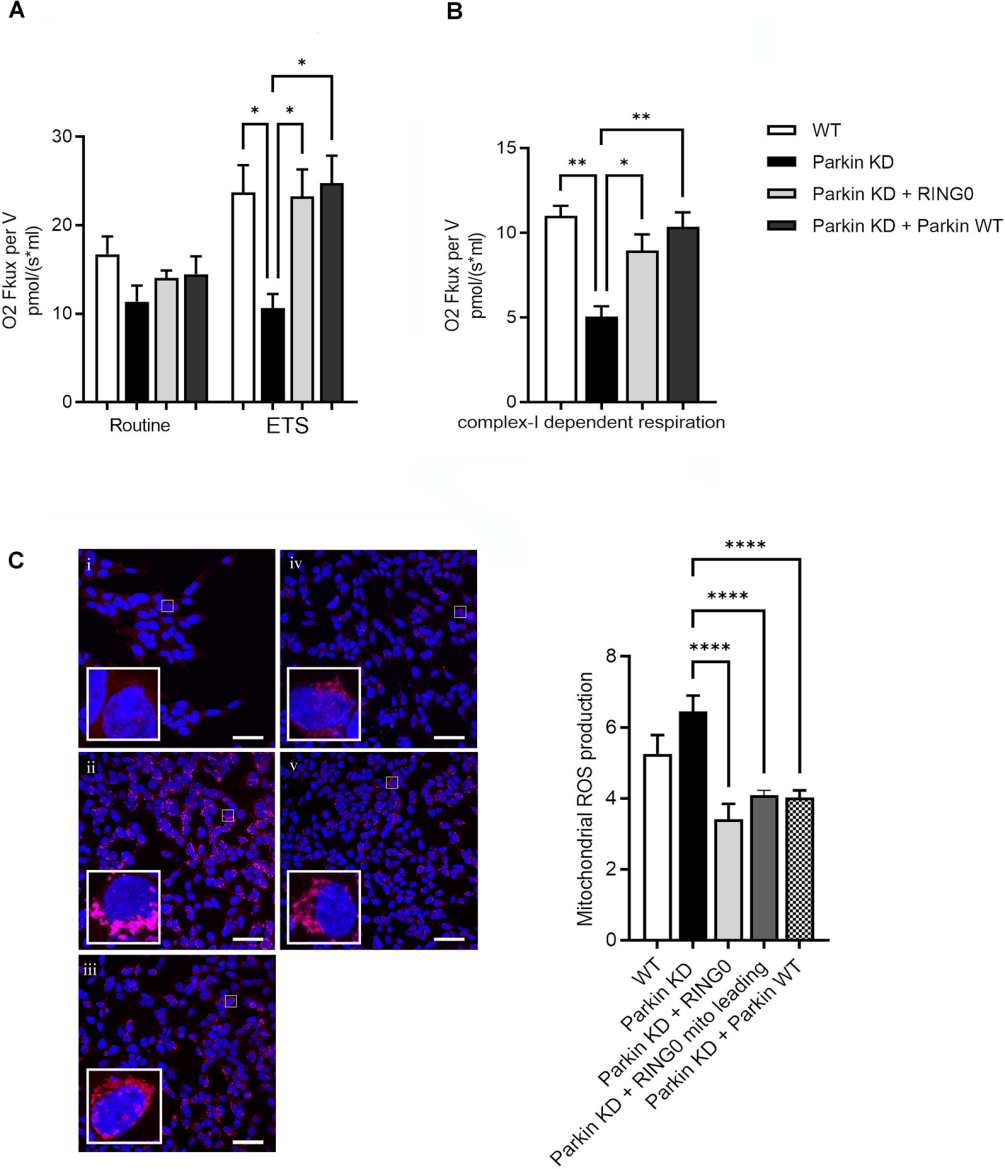
Parkin RING0 expression rescues altered mitochondrial phenotypes in Parkin-deficient cells. **A** Maximum respiratory capacity (ETS) in Parkin knockdown (KD) SH-SY5Y cells was significantly rescued after overexpression of Parkin RING0 and full-length Parkin (Parkin WT). Statistical differences were calculated by one-way ANOVA followed by Tukey's post hoc test to correct for multiple comparisons *p ≤ 0.05. **B** Complex I—dependent respiration was significantly restored after overexpression of Parkin RING0 and Parkin WT in Parkin KD SH-SY5Y cells. Statistical differences were calculated by one-way ANOVA followed by Tukey’s post hoc test to correct for multiple comparisons *p ≤ 0.05, **p ≤ 0.01. **C** Mitochondrial ROS levels were significantly reduced after overexpression of Parkin RING0 (iii), RING0 conjugated with a mitochondrial leading sequence (iv), and Parkin WT (v) in Parkin KD (ii) SH-SY5Y cells. WT SH-SY5Y cells are shown in panel (i). MitoSox Red mitochondrial superoxide staining is shown in red. Scale bar: 50 µm. Statistical differences were calculated by one-way ANOVA followed by Tukey’s post hoc test to correct for multiple comparisons ****p ≤ 0.0001

We have previously shown a significant reduction of complex I activity in Parkin-deficient cells [36]. To ascertain the effect on mitochondrial respiration of delivering the isolated RING0 domain to Parkin-deficient cells, we determined the mitochondrial respiratory activity of SH-SY5Y cells with a stable knockdown of Parkin after transfection of Parkin RING0. Mitochondrial respiration was reflected by the oxygen flux under respiratory states of routine and ETS. In addition, complex I-dependent respiration was measured in the same experimental setting after addition of malate, glutamate, and ADP. Parkin knockdown cells displayed a lower FCCP-induced ETS (Fig. 4A) and a reduced complex I-dependent respiration compared to wild type cells (Fig. 4B), which could be restored by expression of the RING0 domain. The transfection of full-length Parkin resulted in a comparable rescue effect. These data suggest that mimicking the binding between the two proteins by delivering the isolated Parkin RING0 domain is sufficient to improve mitochondrial respiratory activity in Parkin-deficient cells.

### Expression of Parkin RING0 domain prevented the generation of excessive reactive oxygen species in Parkin-deficient cells

Since functional abnormalities of the mitochondrial respiration are expected to be associated with changes in mitochondrial-derived reactive oxygen species (mROS) production, we measured mROS levels as a readout of mitochondrial stress. MitoSox Red fluorescence intensity in wildtype cells was overall lower as compared to Parkin-deficient SH-SY5Y cells, which exhibited MitoSox Red-positive mitochondria widely distributed throughout the cytosol (Fig. 4C). Notably, Parkin knockdown cells transfected with the Parkin RING0 domain, the Parkin RING0 domain linked to a mitochondrial leading sequence or full-length Parkin, displayed a reduced MitoSox Red staining, pointing to a rescue effect for lowering the mROS production via reconstitution of the Parkin-SLP-2 interaction (Fig. 4C).

### A Parkin mini-peptide in the RING0 domain corrects mitochondrial dysfunction upon Parkin deficiency

**Fig. 5:**
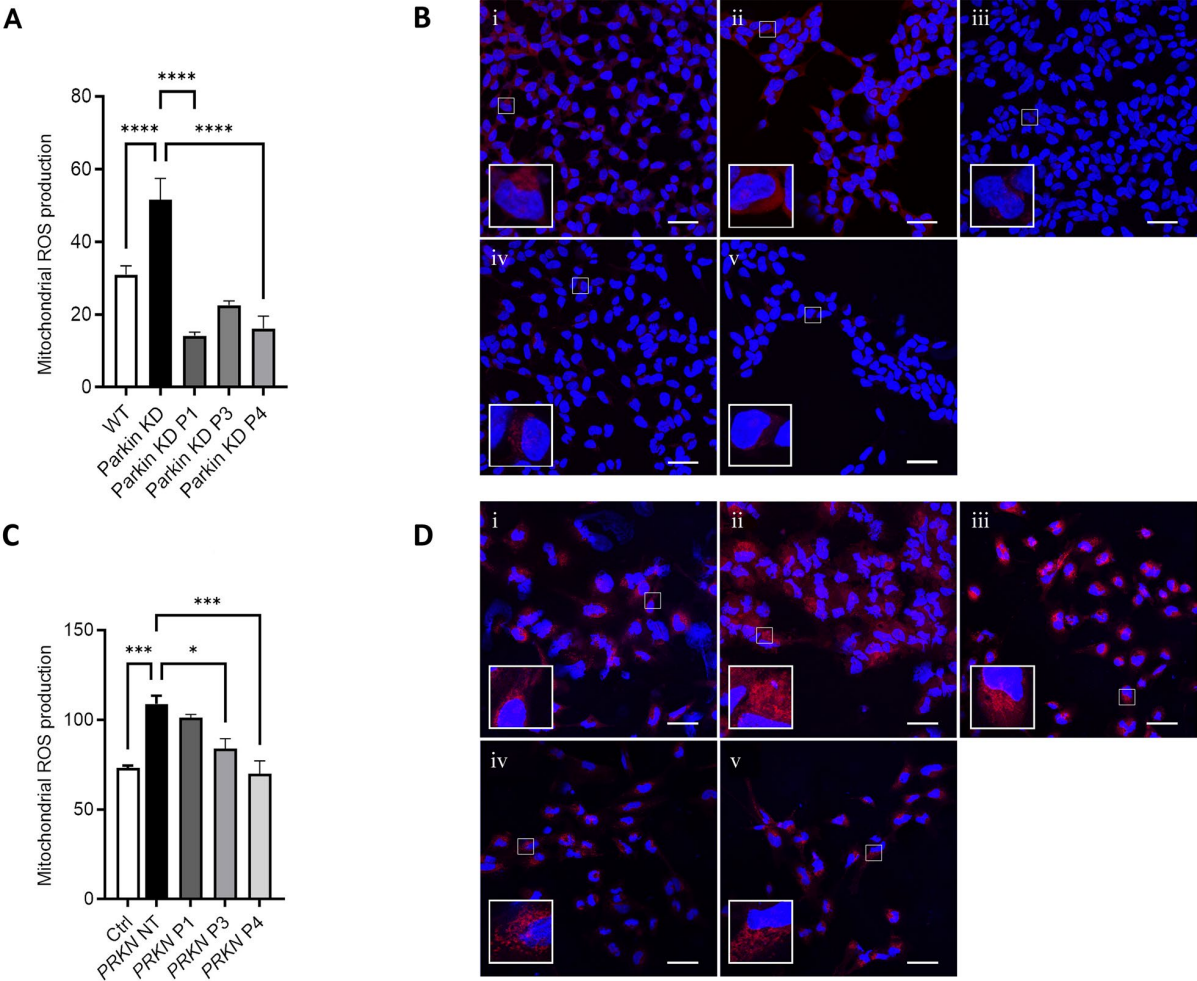
Parkin mini-peptide conjugates rescue altered mitochondrial ROS levels in Parkin-deficient cells. **A**, **B** Delivery of mini-peptide-conjugates #1 (iii), #3 (iv), #4 (v) reduce mitochondrial ROS production in Parkin KD SH-SY5Y cells (ii). (i) WT SH-SY5Y. Statistical differences were calculated by one-way ANOVA followed by Holm-Sidak post hoc test to correct for multiple comparisons ****p ≤ 0.0001. Scale bar: 30 μm. **C**, **D** Mitochondrial ROS production is reduced by mini-peptides #3 (iv) and #4 (v) in hiPSC-derived neurons carrying a *PRKN* mutation (*PRKN* NT, not treated, ii). (i) hiPSC-derived neurons of a control individual; (iii) mini-peptide #1 in hiPSC-derived neurons carrying a *PRKN* mutation. Scale bar: 50 μm. Statistical differences were calculated by one-way ANOVA followed by Holm-Sidak post hoc test to correct for multiple comparisons ***p ≤ 0.001, *p ≤ 0.05

Based on these data, we designed a Parkin mini-peptide in a region in RING0 known to harbour natural PD-causing mutations. This was to test whether such a mini-peptide could substitute mutated and/or absent Parkin protein in its ability to bind to mitochondrial SLP-2 and thus also rescue or stabilise mitochondrial functions disrupted by the absence of Parkin. The mini-peptide was tested for its solvent accessibility in the RING0 domain and in the multidomain Parkin structure. The engineered Parkin mini-peptide was attached to three different conjugates (#1: TAT sequence, #3: cell-permeable peptide able to enter mitochondria, #4: mitochondrial transporter), and its effect on mitochondrial dysfunction associated with Parkin deficiency was investigated in Parkin-deficient SH-SY5Y cells. Remarkably, increased mROS levels in Parkin-deficient cells were reduced after 16 h treatment with the Parkin mini-peptide attached to all three conjugates (#1, #3, and #4) (Fig. 5A). For one of the peptide conjugates, we detected also a significant improvement of a respirometry phenotype in Parkin-deficient SH-SY5Y cells (Additional file 1: Fig. S4). Application of the same concentrations of the peptide-conjugates for 3 h did not result in any change of the phenotypes measured but started to show measurable effects after 8 h of exposure (Additional file 1: Fig. S5). Fluorescence imaging of a GFP-tagged Parkin mini-peptide (#1) confirmed the presence of the peptide and its stable quantity in the cytoplasm after 16 h of application in the cultures (Additional file 1: Fig. S6).

We also tested the three synthesized versions of the Parkin mini-peptide (#1, #3, #4) in hiPSC-derived neurons derived from a PD patient carrying a homozygous deletion of exon 3 in the *PRKN* gene, as a more disease-relevant cellular model. Peptide-conjugates #3 and #4 achieved a significant rescue of the mROS levels in this neuronal model system harboring an endogenous *PRKN* mutation (Fig. 5B). Accordingly, hiPSC-derived neurons with an endogenous *PRKN* mutation exhibit significantly lower levels of TMRM fluorescence, indicating a loss of the mitochondrial membrane potential, that could also be recovered by the administration of all three peptide-conjugates (#1, #3, and #4) (Additional file 1: Fig. S7).

## Discussion

Here, we provide evidence that the RING0 domain of Parkin is a region heavily involved in a functional interaction with mitochondrial SLP-2, by combining co-immunoprecipitation and PLA assays with computational modeling based on the crystal structure of Parkin. PD-causing mutations in this domain led to a weakened binding between Parkin and SLP-2. Delivery of the isolated RING0 domain and an engineered Parkin mini-peptide, designed based on its overlap with predicted protein binding sites within the RING0 domain, resulted in the correction of mitochondrial dysfunction observed by several functional measures that include reduced mitochondrial respiration, complex I activity, mROS production and mitochondrial membrane potential in neuroblastoma SH-SY5Y cells and hiPSC-derived neurons with endogenous, disease-causing mutations in *PRKN*.

Loss of Parkin function is the most common known cause of autosomal recessive PD, accounts for 10–20% of early-onset PD in general [9, 67], and it plays a role in sporadic PD [10, 11]. Previous work has shown that one of the molecular hallmarks of Parkin-related PD is a decreased activity of mitochondrial complex I that was identified in various cell and animal models [29, 68, 69]. In support of these data, *PRKN*-knockout mice exhibited a decreased abundance of mitochondrial proteins involved in oxidative phosphorylation, which was accompanied by a reduction in respiratory capacity in striatal mitochondria [30]. We have shown previously that Parkin-depleted SH-SY5Y cells and hiPSC-derived neurons harboring PD-causing *PRKN* mutations display dysfunctional mitochondrial phenotypes including a fragmented mitochondrial morphology and reduced complex I activity [36]. Moreover, we have found that Parkin interacts with mitochondrial SLP-2 [35, 36], which binds to cardiolipin and prohibitins (PHB-1 and PHB-2) to promote the compartmentalization of the mitochondrial inner membrane into cardiolipin-enriched microdomains [37], thus supporting the optimal assembly and function of the electron transport chain complexes [39, 70, 71]. In fact, SLP-2 deficiency was shown to be associated with altered mitochondrial respiration in T cells [39] and impaired respiratory chain super-complex formation [38], whereas upregulation of SLP-2 expression levels translates into enhanced mitochondrial biogenesis and function [37]. SLP-2 was described to also form a large protease complex with the rhomboid protease PARL and the i-AAA protease YME1L, named SPY [40]. Within this complex, SLP-2 supports the recruitment of PINK1 to PARL, thus facilitating the proteolytic cleavage of PINK1, and it limits the PARL-mediated cleavage of PGAM5, an important regulator of mitochondrial respiration, dynamics, and cell survival [72, 73]. Moreover, by negatively affecting the activity of OMA1, SLP-2 stabilizes OPA1, allowing stress-induced mitochondrial hyperfusion [40], thus enabling respiration and protection against mitophagy under starvation conditions [74, 75].

The interaction of Parkin with SLP-2 might be essential for these protective effects under physiological or cellular stress conditions, since selective knockdown of either of these proteins led to reduced mitochondrial and neuronal function in neuronal cells and in *Drosophila*, where a double knockdown led to a further worsening of Parkin-deficiency phenotypes, indicating that SLP-2 activity in Parkin deficiency maintains partial mitochondrial protection. Therefore, we have tested a moderate overexpression of SLP-2 in a Parkin deficiency background, which was able to restore phenotypes of mitochondrial dysfunction in cellular and *Drosophila* models [36].

In this work, we focused on the identification of the critical domain involved in the Parkin-SLP-2 binding and showed that SLP-2 preferentially binds to the N-terminal RING0 domain of Parkin. Far-Western blot and split GFP  analyses showed a direct interaction between the two proteins. Further, PLA analysis indicated that the interaction of overexpressed Parkin RING0 and endogenous SLP-2 occurs at the mitochondria. Although Parkin translocates to the outer mitochondrial membrane (OMM), a direct interaction between Parkin and inner mitochondrial membrane (IMM) proteins was described previously. For instance, it interacts with PHB2, which forms a complex with SLP-2 [76], and with Mitofilin or Mic60, one of the core mitochondrial contact site and cristae organizing system (MICOS) subunits [77], resulting in their ubiquitination. The MICOS complex plays a crucial role in the formation of cristae junctions and contact sites between the outer and inner membranes [78]. Further, a proteomic study detected interactions between phosphatidylglycerophosphate synthase 1 (PGS1), involved in cardiolipin synthesis, Mic60, and SLP-2 [79] as well as a role of SLP-2 in the MICOS assembly by interacting with MIC13, another MICOS complex subunit [80]. These data may explain the interaction between Parkin and SLP-2 at the mitochondria, even though our previous findings suggested that SLP-2 is neither consistently ubiquitinated by Parkin, nor does it affect Parkin recruitment to mitochondria [36].

We aimed to test whether we could restore the abolished/weakened interaction of Parkin and SLP-2 due to Parkin deficiency by cellular delivery of the isolated RING0 domain. Intriguingly, transfection of RING0 could restore compromised mitochondrial respiration and impaired complex I activity caused by Parkin deficiency. Furthermore, increased ROS production observed under these circumstances could be significantly decreased by RING0 transfection. The levels of mitochondrial function rescue achieved by transfection of RING0 were comparable to those achieved by both expression of RING0 coupled to a mitochondrial leading sequence and full-length Parkin. This is in line with the observed partial localization of the isolated Parkin RING0 domain in the vicinity of mitochondria, despite the fact that Parkin translocation to the outer membrane of depolarized mitochondria requires the active site cysteine C431, which is part of the RING2 domain [81].

By computational modeling, we identified potential binding sites for SLP-2 within the Parkin RING0 domain, and among them, three naturally occurring disease-causing missense mutations within these predicted binding regions interfere substantially with the binding to SLP-2 as shown by co-immunoprecipitation and PLA. Therefore, in this region we designed a mini-peptide that might thus be capable of mimicking Parkin binding to SLP-2 enough to boost SLP-2 function. The Parkin mini-peptide was synthesized with three different conjugates: a cell permeant TAT-sequence (#1) to allow intracellular delivery of protein cargos, which was used previously to also deliver mitochondrial peptides [52], and two cell-permeable sequences (#3 and #4) shown previously to enable transport to mitochondria [53, 54]. Notably, treatment with both Parkin mini-peptide-conjugate #3 and #4 decreased mitochondrial ROS levels in both Parkin-deficient SH-SY5Y cells and hiPSC-derived neurons harboring disease-causing *PRKN* mutations. The conjugates used for Parkin mini-peptide #3 and #4 have already been shown to be biocompatible and not to affect mitochondrial functionality [53, 54]. For the Parkin mini-peptide-conjugate #1, a reduction of mitochondrial ROS production was observed only in the SH-SY5Y cells, possibly reflecting a less stable delivery of the Parkin peptide by this conjugate to the mitochondrial compartment. Thus, our mini-peptide-approach, aimed at testing the capability to substitute and/or restore the binding and stabilization of SLP-2 in Parkin-deficient cells, did promote correct mitochondrial functionality, and these in vitro data thus provide the basis for further in vivo validation of this approach. Interestingly, a previous study has developed a cell-permeable full-length Parkin protein displaying a high level of cell permeability, which also maintained the E3 ubiquitin ligase activity of native Parkin. Delivery of full-length Parkin restored mitochondrial function and protected against neuronal toxicity in neuronal cells and animals [82, 83].

Macromolecular therapeutics, including proteins and peptides, can target specific molecular pathways and modulate biological responses by regulating protein interactions and might therefore be used in the treatment of a wide range of diseases [42, 43, 84]. However, macromolecules often have the disadvantage of having little bioavailability, and they require delivery vehicles to enter cells. Cell-penetrating peptides, including the HIV TAT transduction sequence used in this study, have been successfully used to deliver protein cargos into cells and allow systemic protein delivery into a variety of tissues including the brain [42, 82, 85, 86]. Specific consensus amino acid sequences are frequently involved in protein interactions [84], and therefore, biologically active intracellular peptides can be used to inhibit or restore specific protein interactions involved in relevant pathological processes [44]. Such an approach might be more targeted as compared to expressing a full-length protein as it only modulates a specific function of the protein of interest. In addition, peptide drugs develop less immunogenicity, they are easier to generate in a pure form, and have a lower production cost [87–89]. Further, compared to conventional small molecule drugs, a peptide-based approach is characterized by a higher specificity and fewer off-target effects [44]. Thus, the generation of a peptide containing the Parkin minimal region involved in the Parkin-SLP-2 interaction and its intracellular targeting to the mitochondria could potentially lead to the development of organelle-specific therapeutics for restoring correct mitochondrial function in Parkin-linked PD. Such a technology might represent a valid alternative to genetic engineering to correct *PRKN* gene mutations or to activating Parkin with small molecule drugs. An important drawback of peptide drugs is however their poor in vivo stability with a short half-life due to their inherent chemical properties [90]. These challenges can be overcome by different strategies, which can be applied to improve the physicochemical properties, for example chemical modification of the sequence to stabilize the peptide secondary structure and appropriate conjugation to stability enhancers [91]. Notably, while we have not systematically investigated the half-life of the Parkin mini-peptide, we observed a consistent effect after 16 h of application, when the peptide was still present in the cell cultures. To test the bioavailability and the pharmacodynamic profile, the peptide itself or a mimetic form will need to be tested in in vivo studies, and the lack thereof represents a limitation of our study. Interestingly, it was suggested that a small mitochondria-targeted tetrapeptide, Elamipretide/MTP-131, is able to ameliorate mitochondrial dysfunction in different model systems of a range of diseases, including PD and neurodegeneration [92, 93]. This tetrapeptide can cross the blood–brain barrier [94, 95], entering neural cells, where it is absorbed by mitochondria and binds to the inner mitochondrial membrane phospholipid cardiolipin [96, 97]. The neuroprotective effects of Elamipretide are mediated by inhibition of neural oxidative stress, neuroinflammation, toxic protein accumulation, and neural apoptosis [93]. Subcutaneous injections of this tetrapeptide have been tested in a phase-3 clinical trial for mitochondrial myopathy (https://www.clinicaltrials.gov/study/NCT03323749) demonstrating that it is well-tolerated but could not improve the outcome [98]. Clinical studies are now required to assess the pharmacokinetics and pharmacodynamics of elamipretide and other mitochondria-targeted peptides in patients with neurodegenerative diseases, including PD.

## Conclusions

Our findings suggest that the interaction between Parkin and SLP-2 is important to prevent accumulation of dysfunctional mitochondria. Furthermore, the delivery of Parkin RING0 domain or a Parkin mini-peptide within the RING0 domain involved in this specific protein–protein interaction can rescue compromised mitochondrial function in Parkin-deficient neuroblastoma SH-SY5Y cells and in hiPSC-derived neurons harboring disease-causing *PRKN* mutations. These results might open the way for the development of a Parkin peptide-based drug or peptidomimetic-like therapeutic agents that can reconstitute this specific protein–protein interaction.

### Supplementary Information

**Additional file 1: Table S1.** Primer sequences used to amplify the Parkin domains. **Table S2.** Genotypic and phenotypic information for investigated PD patients with *PRKN* mutations. **Table S3.** Predicted candidate binding sites in Parkin RING0 that overlap with PD-causing mutations. **Figure S1.** The interaction between Parkin and SLP-2 is detected by using the Tripartite split GFP assay in *E. coli.* **Figure S2.** The transfected RING0 domain localises partially to the mitochondria. **A)** HeLa cells were transfected with RING0 with mitochondrial targeting sequence (green: anti-myc; red: anti-GRP75; blue: nuclei) or **B)** with RING0-HA (green: anti-HA; red: anti-GRP75; blue: nuclei). The transfected RING0 domain localizes partially to the mitochondria, whereas the mitochondrial targeting sequence induces complete translocation of RING0 to the mitochondria. Scale bar: 25 µm. **Figure S3.** Overexpressed RING0 domain and its mutants in HeLa cells are present at the mitochondria. Wildtype and mutant RING0 domains (carrying either a P153A, K161A, or K211A mutation) are present at the mitochondria (green) and to a very low extend at the lysosomes (blue). green: GRP75; blue: GFP-LC3b; red: HA-RING0 and HA-RING0 mutants; white: nuclei. Scale bar: 10 µm. **Figure S4.** Oxygen consumption impairment in Parkin-deficient cells improves after Parkin mini-peptide application. **(A)** Representative curves of fluorescence signal, generated by the oxygen probe, reflecting dissolved oxygen in the culture medium of WT SH-SY5Y, Parkin KD SH-SY5Y, and Parkin KD SH-SY5Y cells treated with Parkin mini-peptide #1. **(B)** Relative quantification of oxygen consumption. Cellular respiration is significantly increased by mini-peptide #1. Error bars represent the mean ± SEM of 3 data points. Statistical differences were determined using one-way ANOVA followed by Tukey’s post hoc test to correct for multiple comparisons. *p ≤ 0.05, **p ≤ 0.01, ns = not significant. RFU: relative fluorescence units. **Figure S5.** Mitochondrial ROS levels in Parkin-deficient cells after Parkin mini-peptide application for 3 h and 8 h, respectively. Delivery of mini-peptide-conjugates #1 (iii), #3 (v), #4 (vii) are not able to reduce mitochondrial ROS production in Parkin KD SH-SY5Y cells (ii) after application for 3 h. (i) WT SH-SY5Y cells. After 8 h of peptide application, a significant effect is detected for mini-peptide-conjugates #3 (vi) and #4 (viii). Statistical differences were calculated by one-way ANOVA followed by Holm-Sidak post hoc test to correct for multiple comparisons. ** p ≤ 0.01, **** p ≤ 0.0001. Scale bar: 50 μm. **Figure S6.** Detection of Parkin mini-peptide marked with a GFP-tag. SH-SY5Y cells were treated with Parkin mini-peptide (#1) and mitochondria stained with MitoTracker red fluorescent dye. Live cell imaging detected the Parkin mini-peptide in SH-SY5Y cells 3 h **(A)** and 16 h after application **(B)**, respectively. red: MitoTracker red; green: GFP-Parkin mini-peptide #1. Scale bar: 50 µm. **Figure S7.** Parkin mini-peptide conjugates rescue altered mitochondrial membrane potential levels (Δψ) in hiPSC-derived neurons carrying endogenous *PRKN* mutations. Mitochondrial membrane potential is increased by mini-peptides #1 (C), #3 (D), and #4 (E) in hiPSC-derived neurons carrying *PRKN* mutations (*PRKN* NT, not treated, B). hiPSC-derived neurons of control individuals (A). Scale bar: 20 μm. Statistical differences were calculated by one-way ANOVA followed by Holm-Sidak post hoc test to correct for multiple comparisons **** p ≤ 0.0001, *** p ≤ 0.001, ** p ≤ 0.01, * p ≤ 0.05.

## Data Availability

All data generated during this study are included in this article and its supplementary information files.

## Abbreviations

PD: Parkinson’s disease
SLP-2: Stomatin-like protein 2
hiPSCs: Human induced pluripotent stem cells
PLA: Proximity ligation assay
DA: Dopaminergic
Ubl: Ubiquitin-like
UPD: Unique Parkin Domain
IBR: In-Between-RING
REP: Repressor element of Parkin
PPIs: Protein–protein interactions
CCCP: Carbonyl Cyanide m-chlorophenylhydrazone
SDS-PAGE: Sodium dodecylsulfat polyacrylamid gel electrophoresis
RT: Room temperature
mito-GFP: GFP-tagged mitochondrial leading sequence
lyso-GFP: GFP-tagged LC3b
IMAC: Immobilized-metal affinity chromatography
ETS: Maximal capacity of the respiratory chain
FCCP: Carbonyl cyanide p-(trifluoromethoxy) phenylhydrazone
ROX: Residual oxygen consumption
mROS: Mitochondrial-derived reactive oxygen species
