# Supplementary material for: Intracellular delivery of Parkin-RING0-based fragments corrects Parkin-induced mitochondrial dysfunction through interaction with SLP-2
Source: J Transl Med. 2024 Jan 16;22:59. doi: 10.1186/s12967-024-04850-3 (PMC10790385; doi:10.1186/s12967-024-04850-3)
Supplement: Supplementary file 1 — Additional file 1: Table S1. Primer sequences used to amplify the Parkin domains. Table S2. Genotypic and phenotypic information for investigated PD patients with PRKN mutations. Table S3. Predicted candidate binding sites in Parkin RING0 that overlap with PD-causing mutations. Figure S1. The interaction between Parkin and SLP-2 is detected by using the Tripartite split GFP assay in E. coli. Figure S2. The transfected RING0 domain localises partially to the mitochondria. A) HeLa cells were transfected with RING0 with mitochondrial targeting sequence (green: anti-myc; red: anti-GRP75; blue: nuclei) or B) with RING0-HA (green: anti-HA; red: anti-GRP75; blue: nuclei). The transfected RING0 domain localizes partially to the mitochondria, whereas the mitochondrial targeting sequence induces complete translocation of RING0 to the mitochondria. Scale bar: 25 µm. Figure S3. Overexpressed RING0 domain and its mutants in HeLa cells are present at the mitochondria. Wildtype and mutant RING0 domains (carrying either a P153A, K161A, or K211A mutation) are present at the mitochondria (green) and to a very low extend at the lysosomes (blue). green: GRP75; blue: GFP-LC3b; red: HA-RING0 and HA-RING0 mutants; white: nuclei. Scale bar: 10 µm. Figure S4. Oxygen consumption impairment in Parkin-deficient cells improves after Parkin mini-peptide application. (A) Representative curves of fluorescence signal, generated by the oxygen probe, reflecting dissolved oxygen in the culture medium of WT SH-SY5Y, Parkin KD SH-SY5Y, and Parkin KD SH-SY5Y cells treated with Parkin mini-peptide #1. (B) Relative quantification of oxygen consumption. Cellular respiration is significantly increased by mini-peptide #1. Error bars represent the mean ± SEM of 3 data points. Statistical differences were determined using one-way ANOVA followed by Tukey’s post hoc test to correct for multiple comparisons. *p ≤ 0.05, **p ≤ 0.01, ns = not significant. RFU: relative fluorescence units. Figure S5. Mitochondrial ROS [file 12967_2024_4850_MOESM1_ESM.docx]

**Additional Material**

**Table S1.** Primer sequences used to amplify the Parkin domains.

| **Parkin fragments** | **amino acids** | **primers** |
| --- | --- | --- |
| N-terminus | 1-224 | 5’-GAGCTAGCCACCATGATAGTGTTTGTCAGG-3’  5’-GTGAATTCCTAGTCAGTGTGCAGAATGAC-3’ |
| C-terminus | 225-465 | 5’-GGGTCGACCGCTTTGCACCTGATCGCA-3’  5’-GTGAATTCCTAGTCAGTGTGCAGAATGAC-3’ |
| UBL domain | 1-76 | 5`-GGGTCGACCATGATAGTGTTTGTCAG-3’  5’-TTGCGGCCGCAACTATTTTCTCCACGGTCTCT-3’ |
| RING0 | 145-224 | 5’-GGGTCGACCATGAGCTTTTATGTGTATTGC-3’  5’-TTGCGGCCGCAACTAAGCTACTGAATGTTTCCTT-3’ |
| RING1 | 225-324 | 5’-GGGTCGACCGCTTTGCACCTGATCGCA-3’  5’-TTGCGGCCGCAACTAGACACACTCCTCTGCACCA-3’ |
| RING2 | 411-465 | 5’-GGGTCGACCATGATCAAGAAAACCACCAAG-3 and 5- TTGCGGCCGCAACTACACGTCGAACCAGTG-3’ |

**Table S2.** Genotypic and phenotypic information for investigated PD patients with *PRKN* mutations.

| **ID** | **Cell type** | **Sex** | **Age at onset (yr)** | **Age at biopsy (yr)** | **PRKN mutation** | **Zygosity** | **Consequence** | **Affection status** |
| --- | --- | --- | --- | --- | --- | --- | --- | --- |
| FFF-040 (FFF0332011; B-1946) | fibroblasts/hiPSC-derived neurons | F | 30 |  | del ex3 | homozygous | frameshift | affected |
| FFF-026 (FFF0072010; S-0949) | fibroblasts | F | 39 |  | c.101_102 del AG (p.Gln34Argfs*4) | homozygous | frameshift at aa 34 | affected |
| DNA10755 | fibroblasts | F | 32 |  | c.101_102 del AG (p.Gln34Argfs*4 and del ex3 and 4 | compound heterozygous | frameshift at aa 34 | affected |
| DNA22026 | fibroblasts | F | 63 |  | del exon 3 and 4 | compound heterozygous |  | affected |
| L5415 | fibroblasts | F | / | 35 | c.823 C>T (p.Arg275Trp); c.1054 T>C (p.Cys352Arg) | compound heterozygous | missense | affected |
| B125 | fibroblasts/hiPSC-derived neurons | M | 43 | 58 | c.971 del T (p. p.Val324Alafs*111) | homozygous | frameshift at aa 324 | affected |
| B11 | fibroblasts | M | 64 | 75 | c.971 del T (p. p.Val324Alafs*111) + del ex7 (p.Arg245Serfs*8) | compound heterozygous | frameshift at aa 324 + frameshift at aa 345 | affected |
| 802 | fibroblasts/hiPSC-derived neurons | F | / | adult | / | / |  | unaffected |
| SFC084-03-02 | hiPSC-derived neurons | F | / | adult | / | / |  | unaffected |

**Table S3.** Predicted candidate binding sites in Parkin RING0 that overlap with PD-causing mutations.

| **Amino acid residue** | **Reference** | **Number of consensus predictions^*^** |
| --- | --- | --- |
| 145 | (1) | 3 |
| 143 | (2) | 2 |
| 180 | (3) | 2 |
| 153 | (4) | 1 |
| 161 | (5) | 1 |
| 171 | (6) | 1 |
| 175 | (7) | 1 |
| 179 | (8) | 1 |
| 211 | (9, 10) | 1 |

The three amino acid residues marked in grey were selected for further mutagenesis experiments.

^*^3: three prediction methods; 2: two prediction methods; 1: one prediction method

Evidence1 Position HGMD2 Site Selection3 Comment


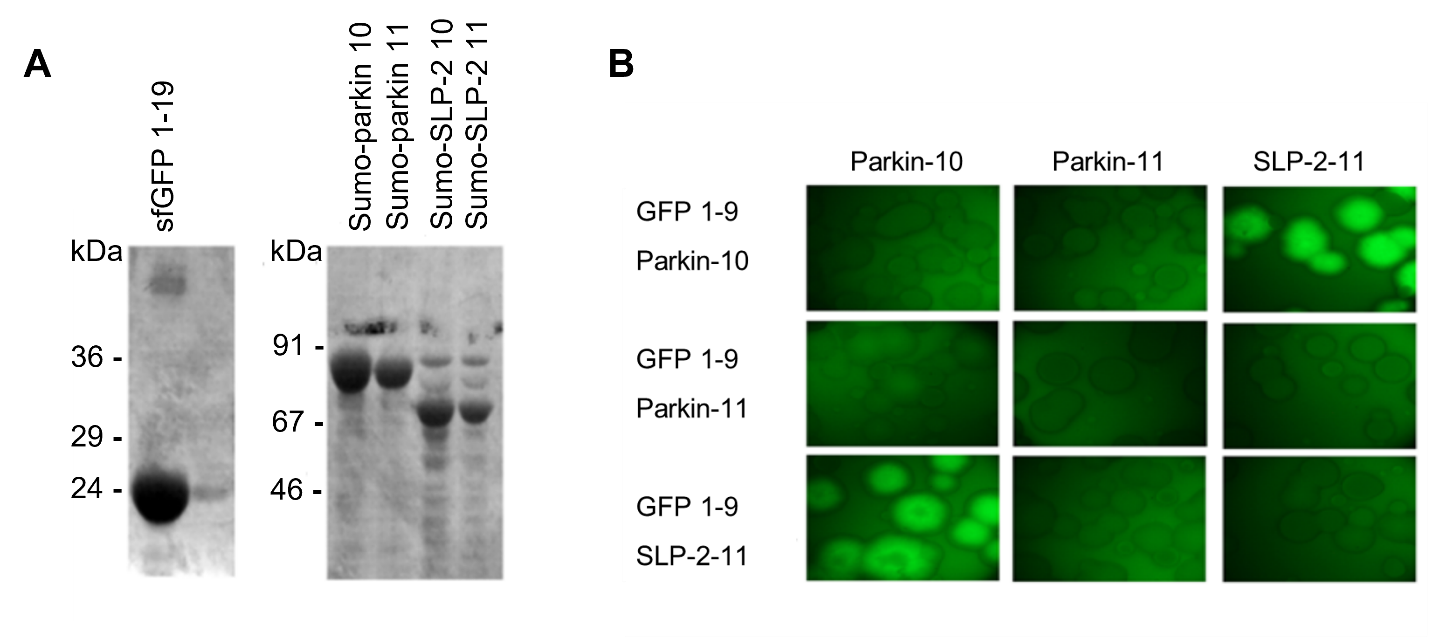


**Fig. S1.** The interaction between Parkin and SLP-2 is detected by using the Tripartite split GFP assay in *E. coli.*

**
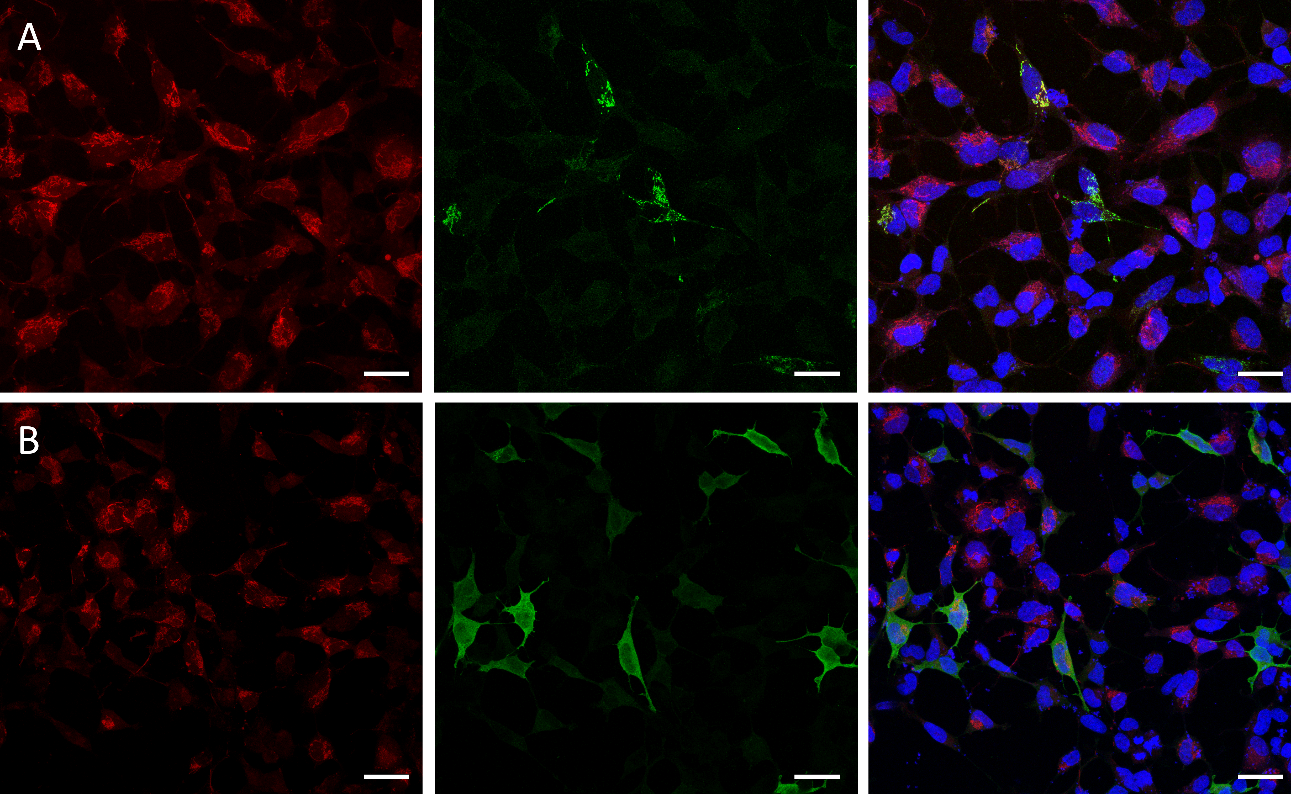
**

**Fig. S2.** **The transfected** **RING0 domain localises partially to the mitochondria. A)** HeLa cells were transfected with RING0 with mitochondrial targeting sequence (green: anti-myc; red: anti-GRP75; blue: nuclei) or **B)** with RING0-HA (green: anti-HA; red: anti-GRP75; blue: nuclei). The transfected RING0 domain localizes partially to the mitochondria, whereas the mitochondrial targeting sequence induces complete translocation of RING0 to the mitochondria. Scale bar: 25 µm.

**Fig. S3.** **Overexpressed RING0 domain and its mutants in HeLa cells are present at the mitochondria.** Wildtype and mutant RING0 domains (carrying either a P153A, K161A, or K211A mutation) are present at the mitochondria (green) and to a very low extend at the lysosomes (blue). green: GRP75; blue: GFP-LC3b; red: HA-RING0 and HA-RING0 mutants; white: nuclei. Scale bar: 10 µm.


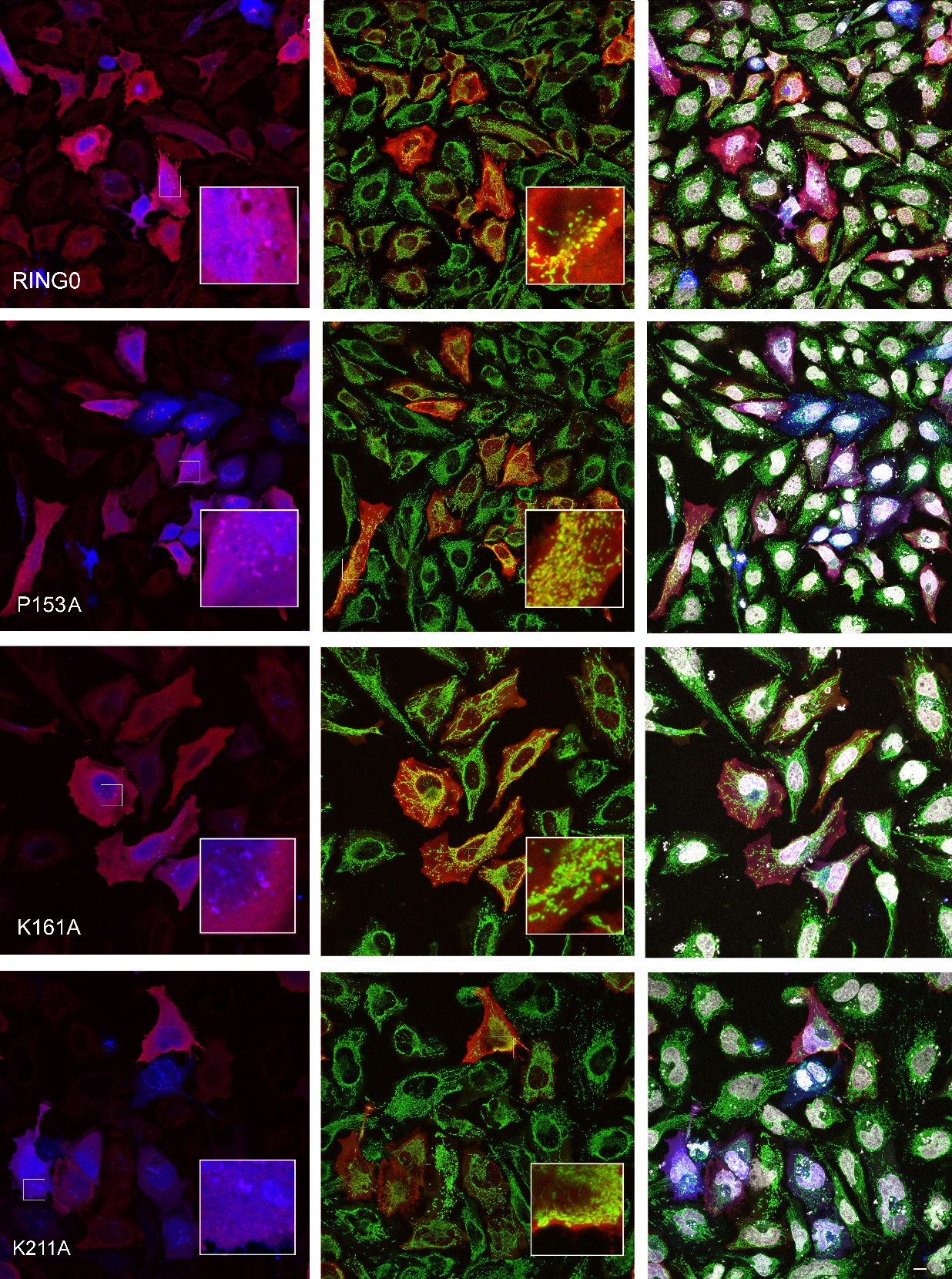


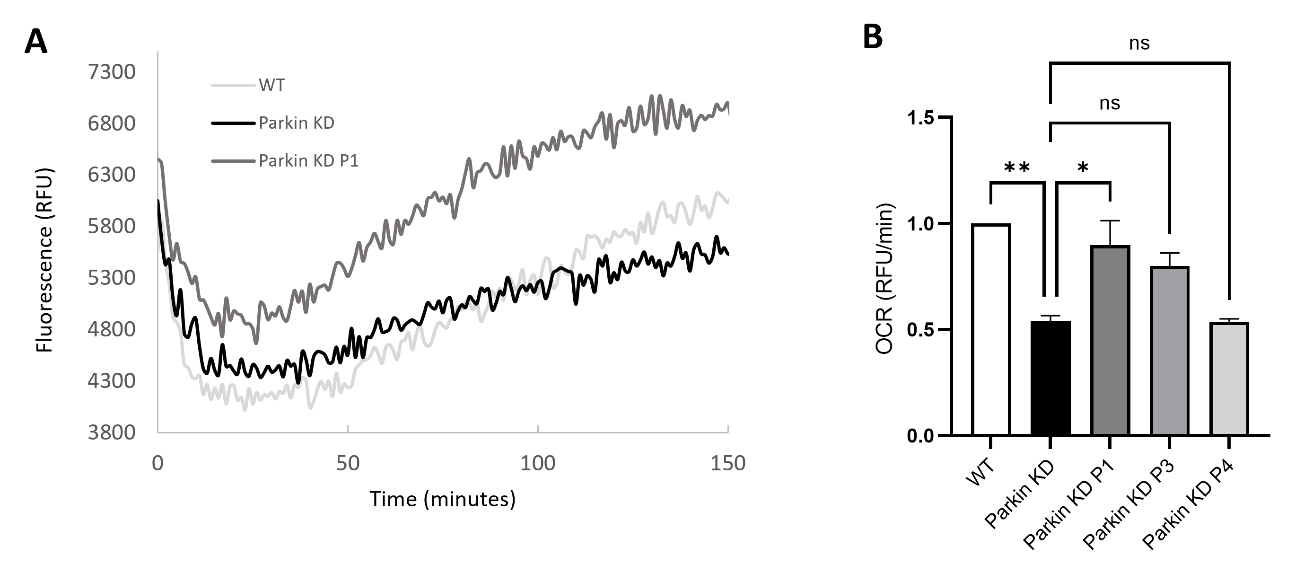


**Fig. S4. Oxygen consumption impairment in Parkin-deficient cells improves after Parkin mini-peptide application. (A)** Representative curves of fluorescence signal, generated by the oxygen probe, reflecting dissolved oxygen in the culture medium of WT SH-SY5Y, Parkin KD SH-SY5Y, and Parkin KD SH-SY5Y cells treated with Parkin mini-peptide #1. **(B)** Relative quantification of oxygen consumption. Cellular respiration is significantly increased by mini-peptide #1. Error bars represent the mean ± SEM of 3 data points. Statistical differences were determined using one-way ANOVA followed by Tukey’s post hoc test to correct for multiple comparisons. *p≤0.05, **p≤0.01, ns = not significant. RFU: relative fluorescence units.

**
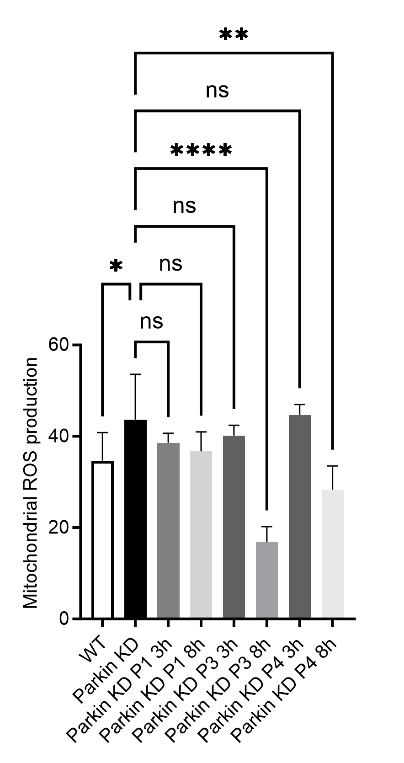
**
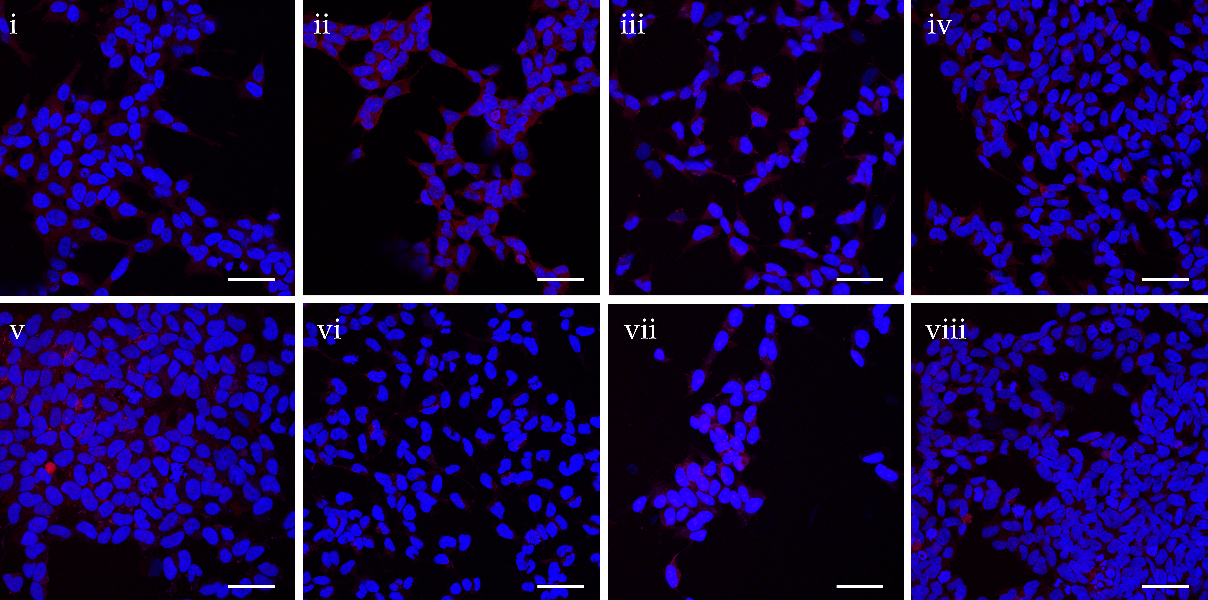
**Fig. S5. Mitochondrial ROS levels in Parkin-deficient cells after Parkin mini-peptide application for 3 h and 8 h, respectively.** Delivery of mini-peptide-conjugates #1 (iii), #3 (v), #4 (vii) are not able to reduce mitochondrial ROS production in Parkin KD SH-SY5Y cells (ii) after application for 3 h. (i) WT SH-SY5Y cells. After 8 h of peptide application, a significant effect is detected for mini-peptide-conjugates #3 (vi) and #4 (viii). Statistical differences were calculated by one-way ANOVA followed by Holm-Sidak *post hoc* test to correct for multiple comparisons. ** p≤ 0.01, **** p≤ 0.0001. Scale bar: 50 μm.

**
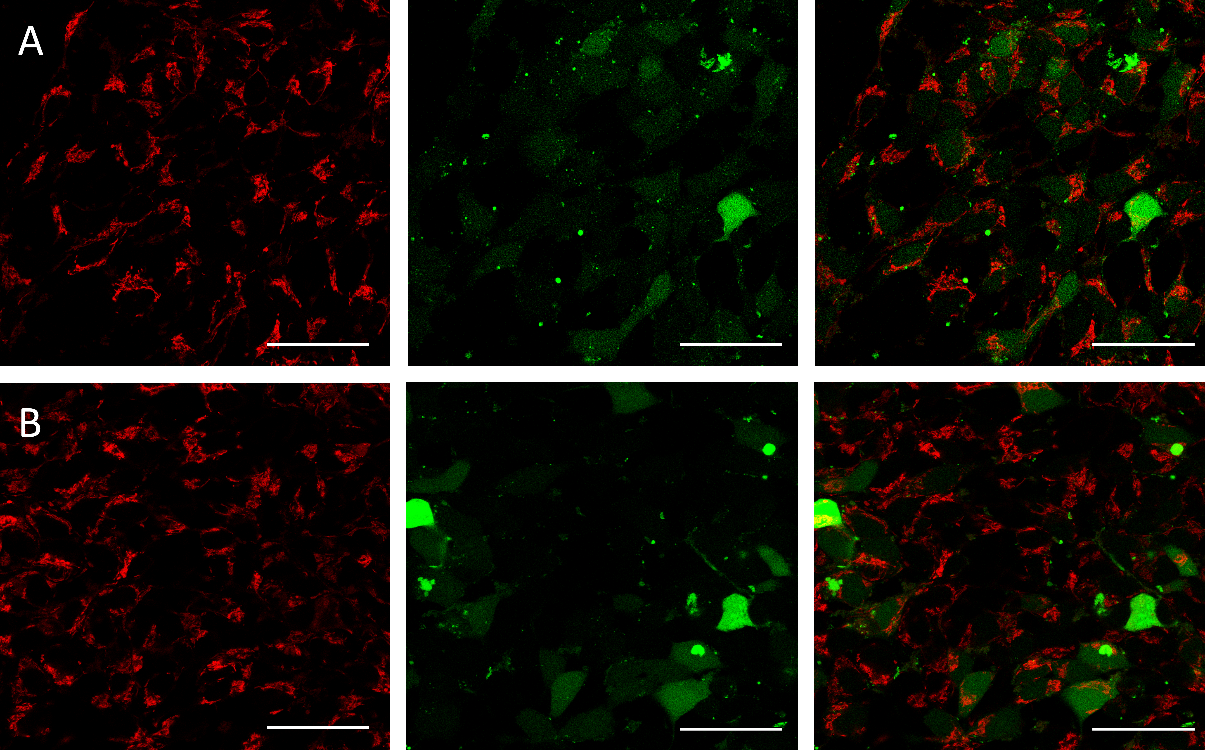
**

**Fig. S6.** **Detection of** **Parkin mini-peptide marked with a GFP-tag.** SH-SY5Y cells were treated with Parkin mini-peptide (#1) and mitochondria stained with MitoTracker red fluorescent dye. Live cell imaging detected the Parkin mini-peptide in SH-SY5Y cells 3 h (A) and 16 h after application (B), respectively. red: MitoTracker red; green: GFP-Parkin mini-peptide #1. Scale bar: 50 µm.

**Fig. S7.** **Parkin mini-peptide conjugates rescue altered mitochondrial membrane potential levels (Δψ) in hiPSC-derived neurons carrying endogenous *PRKN* mutations.** Mitochondrial membrane potential is increased by mini-peptides #1 (C), #3 (D), and #4 (E) in hiPSC-derived neurons carrying *PRKN* mutations (*PRKN* NT, not treated, B). hiPSC-derived neurons of control individuals (A). Scale bar: 20 μm. Statistical differences were calculated by one-way ANOVA followed by Holm-Sidak *post hoc* test to correct for multiple comparisons **** p≤ 0.0001, *** p≤ 0.001, ** p≤ 0.01, * p≤ 0.05.


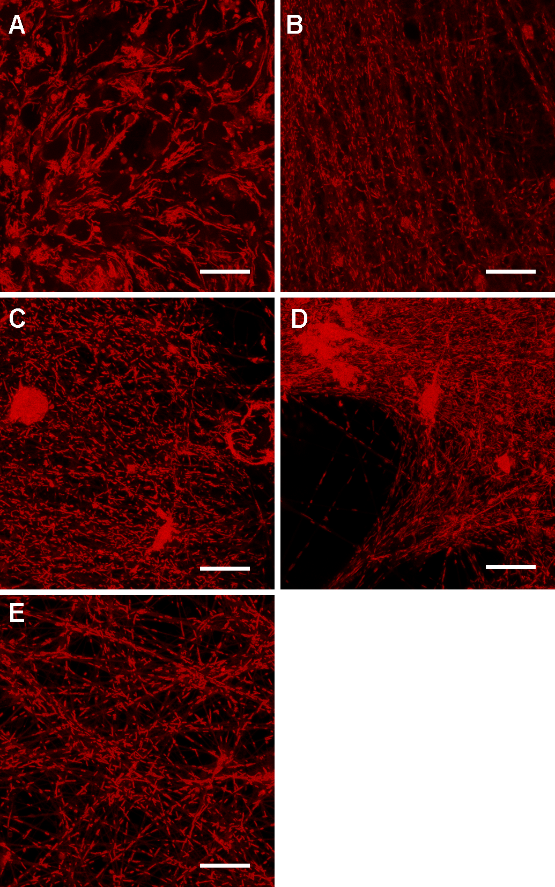

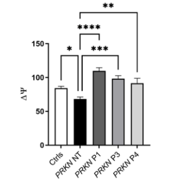


**References**

1. Varrone A, Pellecchia MT, Amboni M, Sansone V, Salvatore E, Ghezzi D, et al. Imaging of dopaminergic dysfunction with [123I]FP-CIT SPECT in early-onset parkin disease. Neurology. 2004;63(11):2097-103.

2. Biswas A, Gupta A, Naiya T, Das G, Neogi R, Datta S, et al. Molecular pathogenesis of Parkinson's disease: identification of mutations in the Parkin gene in Indian patients. Parkinsonism Relat Disord. 2006;12(7):420-6.

3. Chu MK, Kim WC, Choi JM, Hong JH, Kang SY, Ma HI, et al. Analysis of Dosage Mutation in PARK2 among Korean Patients with Early-Onset or Familial Parkinson's Disease. J Clin Neurol. 2014;10(3):244-8.

4. Clark LN, Haamer E, Mejia-Santana H, Harris J, Lesage S, Durr A, et al. Construction and validation of a Parkinson's disease mutation genotyping array for the Parkin gene. Mov Disord. 2007;22(7):932-7.

5. Abbas N, Lucking CB, Ricard S, Durr A, Bonifati V, De Michele G, et al. A wide variety of mutations in the parkin gene are responsible for autosomal recessive parkinsonism in Europe. French Parkinson's Disease Genetics Study Group and the European Consortium on Genetic Susceptibility in Parkinson's Disease. Hum Mol Genet. 1999;8(4):567-74.

6. Brooks J, Ding J, Simon-Sanchez J, Paisan-Ruiz C, Singleton AB, Scholz SW. Parkin and PINK1 mutations in early-onset Parkinson's disease: comprehensive screening in publicly available cases and control. J Med Genet. 2009;46(6):375-81.

7. Funayama M, Li Y, Tsoi TH, Lam CW, Ohi T, Yazawa S, et al. Familial Parkinsonism with digenic parkin and PINK1 mutations. Mov Disord. 2008;23(10):1461-5.

8. Hattori N, Kitada T, Matsumine H, Asakawa S, Yamamura Y, Yoshino H, et al. Molecular genetic analysis of a novel Parkin gene in Japanese families with autosomal recessive juvenile parkinsonism: evidence for variable homozygous deletions in the Parkin gene in affected individuals. Ann Neurol. 1998;44(6):935-41.

9. Periquet M, Lucking C, Vaughan J, Bonifati V, Durr A, De Michele G, et al. Origin of the mutations in the parkin gene in Europe: exon rearrangements are independent recurrent events, whereas point mutations may result from Founder effects. Am J Hum Genet. 2001;68(3):617-26.

10. Lucking CB, Durr A, Bonifati V, Vaughan J, De Michele G, Gasser T, et al. Association between early-onset Parkinson's disease and mutations in the parkin gene. N Engl J Med. 2000;342(21):1560-7.
